# Existence and uniqueness of well-posed fractional boundary value problem

**DOI:** 10.1371/journal.pone.0303848

**Published:** 2024-05-28

**Authors:** Yuanheng Wang, Barrira Jurrat, Muddasir Ejaz, Muhammad Azeem, M. I. Elashiry

**Affiliations:** 1 Mathematics Department of Humanities College, Zhejiang Guangsha Vocational and Technical University of Construction, Dongyang, Jinhua, China; 2 Department of Mathematics, University of Central Punjab, Lahore, Pakistan; 3 Department of Mathematics, Riphah International University, Lahore, Pakistan; 4 Department of Mathematic, Faculty of Arts and Science, Northern Border University, Rafha, Saudi Arabia; 5 Department of Mathematics, Faculty of Science, Fayoum University, El-Fayoum, Egypt; Lahore School of Economics, PAKISTAN

## Abstract

In this paper, the existence and uniqueness of solution for a fractional differential model involving well-posed boundary conditions and implicit fractional differential equation is considered. The desired goals are achieved by using Banach contraction principle and Scheafer’s fixed point theorem. To show the results applicability some examples are presented. The basic mathematical concept of well-posed fractional boundary value issues is investigated in this study. It dives into the existence and uniqueness of these difficulties, offering light on the conditions that allow for both practical and singular solutions. This study contributes to a better knowledge of fractional calculus and its applications in a variety of scientific and technical areas, giving significant insights for both scholars and practitioners.

## 1 Introduction

The specific problem and the constraints placed on it determine if there are solutions to fractional boundary value problems and whether they are unique. I can, however, provide you some general information regarding the well-posedness of problems involving fractional boundary values.

Differential equations with fractional derivatives are used in fractional boundary value problems. The fractional derivative of a function is a non-integer order extension of the classical derivative. Modelling phenomena exhibiting non-local behaviour and long-range interactions frequently makes use of these kinds of issues [1, 2].

An explanation of the issue A fractional differential equation and boundary conditions that specify how the solution should behave at the domain’s edges are often used to solve a fractional boundary value problem. The issue must be clearly stated and be sound mathematically.

One must take into account various factors in order to judge whether a fractional boundary value problem is well-posed:

Existence of solutions: Several techniques, including fixed-point theorems, variational methods, and semi group theory, can be used to prove the existence of solutions. The particular situation at hand and the relevant fractional operator’s characteristics determine the method to be used.

Uniqueness of solutions: Compared to classical differential equations, fractional boundary value issues frequently provide more difficulties in proving the uniqueness of solutions. It can call for further presumptions or certain characteristics of the situation. Under the right circumstances, a variety of methods, such as integral equations, fractional calculus, or comparison principles, can be employed to demonstrate uniqueness.

Stability and regularity: Another aspect of well-posedness is the solution’s consistency and stability. Regularity is concerned with the solution’s smoothness or differentiability, whereas stability is concerned with the solution’s sensitivity to changes in the initial or boundary data.

It is significant to highlight that research on the well-posedness of fractional boundary value problems is ongoing, and the outcomes depend on the particular problem and the fractional order at play. In comparison to classical calculus, the area of fractional calculus is still developing and many elements are still being researched.

In a wide range of mathematical problems, the existence of a solution is equivalent to the existence of a fixed point for a suitable map. The existence of a fixed point is therefore of paramount importance in several areas of mathematics and other sciences. Existence and uniqueness theorems are useful to find one or more conditions which show that there is exactly one solution to given problem. The existence and uniqueness is proved using fixed point theorems which means that the given fractional differential equations has one fixed point under some suitable conditions on fractional differential equations. Fixed points are useful because many problems in mathematics can be formulated in terms of the existence of a fixed point, and it’s often much easier to show that such points exist and then to approximate them (e.g. numerically) than it is to actually find them explicitly.

Fractional differential equations have more importance in practical field due to its applications in science and engineering such as electrochemistry, fluid flow, rheology, diffusive transport, electrical networks, probability, viscoelasticity, control image, signal processing, biophysics, and electromagnetic theory etc [3–9]. Many researchers worked on the applications of fractional differential equations such as Rousan *et al*. [10] suggested a fractional differential equation that combines the simple harmonic oscillations of an LC circuit with the discharging of an RC circuit. The concept of fractional derivatives is employed in the formulation of a stress-stain relationship for elastomers by Koh and Kelly [11]. They also developed numerical schemes for the dynamic analysis of a single degree of freedom fractional oscillator in the time domain. From last few years existence and uniqueness of boundary value problem (BVP) of corresponding FDEs have been attracted the interest of many researchers. Many mathematicians such as Ahmad *et al*. worked on existence of Caputo type fractional differential equations with four-point non-local fractional integral boundary conditions [12]. Benchohra and Ouaar [13] investigated the existence of solutions for FDEs with integral conditions. Ntouyas worked on existence of first order boundary value problems for fractional differential equations and inclusions with fractional integral boundary condition (BC) [14]. Chai established the existence results of positive solutions for boundary value problems of fractional differential equations [15].

Ahmad and Nieto studied model containing R-L(Riemann-Liouville) fractional integro-differential equations with fractional nonlocal integral BC [16]. Zhong and Lin [17] established the solvability results for a FDM (fractional differential model) having nonlocal and multi-point BVP for FDEs of order 1 < *γ* ≤ 2. Lakoud and Khaldi [18] established sufficient conditions for the existence and uniqueness of solutions of fractional differential equations with fractional integral condition, involving the Caputo fractional derivative. Choudhary and Daftar [19] established existence and uniqueness results on fractional differential model which contains a non-linear multi-order FDE and periodic and anti-periodic boundary conditions. They used green function for finding their results.

Akram and Jurrat [20] presented the existence and uniqueness of three point BVP of non-integer order *β* ∈ [2, 3] which includes M-L (Mittag-Lefer) function. They found the solution of considered BVP in term of M-L function using Caputo fraction derivative then check the existence and uniqueness of solution using fixed point theorems.

Many other researchers worked on existence and uniqueness of fractional differential equations using different definitions of fractional derivatives [21–27]. In parallel to these studies, many other analytical and numerical methods have been proposed in [28–38].

This paper contains a fractional differential model which involves an implicit FDE and well-posed boundary conditions such as:

$$\begin{array}{c} \left\{ \begin{array}{c} C^{\gamma }u\left(t\right)=g\left(t,u\left(t\right),C^{\zeta }u\left(t\right)\right),\;\;\;\;\;3<\gamma \leq 4,\;\;\;\;0<\zeta <1\\ u^{\prime }\left(0\right)=0,\;\;u\left(0\right)=0,\;\;u\left(1\right)=\xi u\left(\mu \right),\;\;\;\mu \in \left(0,1\right),\;\;u^{\prime }\left(1\right)=\lambda J^{\eta }u\left(\nu \right),\;\;\;\nu \in \left(0,1\right), \end{array}\right. \end{array}$$ (1)

where, *γ*, *ζ* are the order of Caputo fractional derivatives, *t* ∈ [0, 1], $0<\xi <\frac{1}{\mu^{2}}$ and $0<\lambda <\frac{1}{\nu^{2}}$. λ, *ξ*, *ν*, *μ* these are parameters on which boundary conditions depends. *J*^*ζ*^ is an Riemann-Liouville fractional integral operator of order *ζ*. And *C*^*γ*^ and *C*^*ζ*^ are Caputo derivatives. We will use Caputo derivative due to its main advantage which is that the initial and boundary conditions for differential equations with the Caputo fractional derivative are analogous to the case of integer order differential equations, so they can be interpreted in the same way. Therefore, it is often used in practical applications.

The motivation to consider the model (1) is the existence results for three-point boundary value problem for differential equations presented by Mohamed houas and Maamar benbachir. They considered the model

$$\begin{array}{c} \left\{ \begin{array}{c} C^{\alpha }x\left(t\right)+g\left(x\left(t\right),C^{\xi }x\left(t\right)\right)=0,t\in J\\ x\left(0\right)=x_{0},\;\;x^{\prime }\left(0\right)=0,\;\;x^{\prime }\left(1\right)=\lambda J^{\sigma }x\left(\eta \right),\;\;\;\eta \in \left(0,1\right), \end{array}\right. \end{array}$$ (2)

where, *α* ∈ (2, 3], *ξ* ∈ (1, 2], *η* ∈ (0, 1), *σ* ∈ [0, 1] and *C*^*α*^ and *C*^*ξ*^ are Caputo derivatives of orders *α* and *ξ* respectively [39]. *J*^*σ*^ is R-L fractional integral operator of order *σ*. Moreover, *η* is a parameter on which boundary condition *x*′(1) = λ*J*^*σ*^*x*(*η*) depends.

Houas and benbachir worked for the fractional differential model involving fractional order *α* ∈ (2, 3] but they did not discuss about the the fractional differential model of order (3,4], this paper will fill this gab.

The solution of a fractional differential equation depends upon different types boundary conditions or initial conditions. Our boundary conditions are well-posed which mean the solution behaviour changes continuously with boundary condition and solution is not highly sensitive to changes in final data. Solution of well-posed boundary condition problem can be deducted on a computer using stable algorithm. On the other hand for ill-possed (not well-posed) problem one have to reformulate the algorithm for numerical analysis. There are lot of applications of the fourth order ordinary differential problem in literature such as nonlinear models of suspension bridge [40]. Our model help to convert such type of forth order differential problems in fractional differential model so that the problem will became more accurate as compared to the ordinary differential problem.

This paper is organized as follows: In Section 3, some useful definitions and Lemmas of fractional calculus are presented. The general solution of four-point BVP is described in Section 4. In Section 5, the existence of BVP (1) is proved by Scheafer fixed point theorem. In the same Section uniqueness of BVP (1) is proved by Banach contraction principle.

## 2 Preliminaries

**Definition 2.1**: [3] The fractional derivative of *f* ∈ *C*[0, ∞) in the Caputo sense is defined as:

$$\begin{array}{c} C^{\gamma }u\left(t\right)=\frac{1}{\Gamma (n-\gamma )}\int_{a}^{t}\frac{u^{\left(n\right)}\left(s\right)}{{\left(t-s\right)}^{\gamma +1-n}}ds, \end{array}$$ (3)

where, Γ(.) is the Euler gamma function.

**Definition 2.2**: [11] The R-L fractional integral operator of order *γ* > 0 for a continuous function *f* on [0, ∞) is defined as:

$$\begin{array}{c} J^{\gamma }u\left(t\right)=\frac{1}{\Gamma (\gamma )}\int_{a}^{t}\frac{u(s)}{{\left(t-s\right)}^{1-\gamma }}ds. \end{array}$$ (4)

**Lemma 2.1** [41]. Let *γ* > 0, then the general solution to the fractional differential equation differential *C*^*γ*^*u*(*t*) = 0, is given by *u*(*t*) = *k*_0_ + *k*_1_*t* + *k*_2_*t*^2^ + ….. + *k*_*m*−1_*t*^*m*−1^, *where* $k_{j}\in I\;R$, *j* = 0, 1, 2, …., *m* − 1, *m* = [*γ*] + 1.

**Lemma 2.2** [19] Let *u* ∈ *C*^*m*^[0, 1], *γ* ∈ (*m* − 1, *m*), m∈IN. Then the general solution

$$\begin{array}{c} J^{\gamma }C^{\gamma }u\left(t\right)=u\left(t\right)-\sum_{k=0}^{m-1}\frac{t^{k}}{k!}u^{k}\left(0\right). \end{array}$$ (5)

**Lemma 2.3** [21] If a function is constant then its Caputo fractional derivative is,

$$\begin{array}{c} C_{*}^{\gamma }k=0, \end{array}$$

where, *γ* > 0 with *m* − 1 < *γ* < *m* and Caputo fractional derivative of a power function *g*(*u*) = *u*^*ζ*^ is,

$$C_{*}^{\gamma }u^{\zeta }=\{\begin{array}{ll} 0, & for\;\zeta \in M_{0}\;and\;\zeta <[\gamma ],\\ \frac{\Gamma \left(\zeta +1\right)}{\Gamma \left(\zeta +1-\gamma \right)}y^{\zeta -\gamma }, & for\;\zeta \in M_{0}\;and\;\zeta \geq \left[\gamma \right]\;or\;\zeta \notin M_{0}\;and\;\zeta >\left[\gamma \right], \end{array}$$ (6)

where, *M*_0_ = {0, 1, 2, 3…}.

## 3 General solution of BVP (1)

Observed the following BVP as:

$$\begin{array}{c} \left\{ \begin{array}{c} C^{\gamma }u\left(t\right)=L\left(t\right),\;\;\;,\;\;\;3<\gamma <4,\;\;\;0\leq t\leq 1,\\ u^{\prime }\left(0\right)=0,\;\;u\left(0\right)=0,\;\;u\left(1\right)=\xi u\left(\mu \right),\;\;\;0<\mu <1,\;\;u^{\prime }\left(1\right)=\lambda J^{\eta }u\left(\nu \right),\;\;\;\nu \in \left(0,1\right), \end{array}\right. \end{array}$$ (7)

where, *L* on interval [0, 1] is a absolutely continuous function.

The solution of BVP (7), by using Lemmas (2.1) and (2.2), can be express as:

$$\begin{array}{c} u\left(t\right)=\frac{1}{\Gamma (\gamma )}\int_{0}^{t}{\left(t-s\right)}^{\gamma -1}L\left(s\right)ds-k_{0}-k_{1}t-k_{2}t^{2}-k_{3}t^{3}. \end{array}$$ (8)

Lemma 2.1, implies

$$\begin{array}{c} J^{\eta }u\left(t\right)=\frac{1}{\Gamma (\eta +\gamma )}\int_{0}^{t}{\left(t-s\right)}^{\gamma +\eta -1}L\left(s\right)ds-k_{2}\frac{6t^{\eta +2}}{\Gamma (\eta +3)}-k_{3}\frac{24t^{\eta +3}}{\Gamma (\eta +4)}. \end{array}$$ (9)

Using the BC *u*(0) = 0, *u*′(0) = 0 on Eq (8), leads to *k*_0_ = 0 and *k*_1_ = 0 respectively.

Applying BC *u*(1) = *ξu*(*μ*) and *u*′(1) = *λJ*^*η*^*u*(*ν*) on Eq (8), gives

$$\begin{array}{ccc} k_{2} & = & \frac{a\Gamma \left(\eta +3\right)\left(1-\xi \mu^{2}\right)+2b\left(2-\xi \mu^{2}\right)\Gamma \left(\eta +4\right)\left(1-\xi \mu^{3}\right)}{\left(1-\xi \mu^{2}\right)\Gamma \left(\gamma \right)\left[a\Gamma \left(\eta +3\right)\left(1-\xi \mu^{2}\right)+2b\Gamma \left(\eta +4\right)\left(1-\xi \mu^{3}\right)\right]}\int_{0}^{1}{\left(1-s\right)}^{\gamma -1}L\left(s\right)ds\\ & & -\frac{\xi [a\Gamma \left(\eta +3\right)\left(1-\xi \mu^{2}\right)+2b\left(2-\xi \mu^{2}\right)\Gamma \left(\eta +4\right)\left(1-\xi \mu^{3}\right)]}{\left(1-\xi \mu^{2}\right)\Gamma \left(\gamma \right)\left[a\Gamma \left(\eta +3\right)\left(1-\xi \mu^{2}\right)+2b\Gamma \left(\eta +4\right)\left(1-\xi \mu^{3}\right)\right]}\int_{0}^{\mu }{\left(\mu -s\right)}^{\gamma -1}L\left(s\right)\\ & & +\frac{\Gamma \left(\eta +3\right)\left(1-\xi \mu^{2}\right)\Gamma \left(\eta +4\right)\left(1-\xi \mu^{3}\right)}{\Gamma \left(\gamma -1\right)[a\Gamma \left(\eta +3\right)\left(1-\xi \mu^{2}\right)+2b\Gamma \left(\eta +4\right)\left(1-\xi \mu^{3}\right]}\int_{0}^{1}{\left(1-s\right)}^{\gamma -2}L\left(s\right)ds\\ & & -\frac{\lambda \Gamma \left(\eta +3\right)\left(1-\xi \mu^{2}\right)\Gamma \left(\eta +4\right)\left(1-\xi \mu^{3}\right)}{\Gamma \left(\eta +\gamma \right)[a\Gamma \left(\eta +3\right)\left(1-\xi \mu^{2}\right)+2b\Gamma \left(\eta +4\right)\left(1-\xi \mu^{3}\right]}\int_{0}^{\nu }{\left(\nu -s\right)}^{\eta +\gamma -1}L\left(s\right)ds \end{array}$$ (10)

and,

$$\begin{array}{ccc} k_{3} & = & \frac{\Gamma \left(\eta +3\right)\left(1-\xi \mu^{2}\right)\Gamma \left(\eta +4\right)}{\Gamma \left(\gamma -1\right)[a\Gamma \left(\eta +3\right)\left(1-\xi \mu^{2}\right)+2b\Gamma \left(\eta +4\right)\left(1-\xi \mu^{3}\right)]}\int_{0}^{1}{\left(1-s\right)}^{\gamma -2}L\left(s\right)ds\\ & & -\frac{\lambda \left(1-\xi \mu^{2}\right)\Gamma \left(\eta +4\right)\Gamma \left(\eta +3\right)}{\Gamma \left(\eta +\gamma \right)[a\Gamma \left(\eta +3\right)\left(1-\xi \mu^{2}\right)+2b\Gamma \left(\eta +4\right)\left(1-\xi \mu^{3}\right)]}\int_{0}^{\nu }{\left(\nu -s\right)}^{\eta +\gamma -1}L\left(s\right)ds\\ & & +\frac{2b\Gamma (\eta +4)}{\Gamma \left(\gamma \right)[a\Gamma \left(\eta +3\right)\left(1-\xi \mu^{2}\right)+2b\Gamma \left(\eta +4\right)\left(1-\xi \mu^{3}\right)]}\int_{0}^{1}{\left(i-s\right)}^{\gamma -1}L\left(s\right)ds\\ & & -\frac{2\xi b\Gamma (\eta +4)}{\Gamma \left(\gamma \right)[a\Gamma \left(\eta +3\right)\left(1-\xi \mu^{2}\right)+2b\Gamma \left(\eta +4\right)\left(1-\xi \mu^{3}\right)]}\int_{0}^{\mu }{\left(\mu -s\right)}^{\gamma -1}L\left(s\right)ds, \end{array}$$ (11)

where, *a* = 6λ*ν*^*η*+3^ − Γ(*η* + 4) and *b* = λ*ν*^*η*+2^ − Γ(*η* + 3).

Putting the values of *k*_0_, *k*_1_, *k*_2_ and *k*_3_ in (8), gives:

*u*(*t*) =

$$\begin{array}{ccc} & & \frac{1}{\Gamma (\gamma )}\int_{0}^{t}{\left(t-s\right)}^{\gamma -1}L\left(s\right)ds-\\ & & \frac{\left[a\Gamma \left(\eta +3\right)\left(1-\mu^{2}\xi \right)+2b\{\left(2-\mu^{2}\xi \right)\left(1-\xi \mu^{3}\right)+t\left(1-\xi \mu^{2}\right)\}\Gamma \left(\eta +4\right)\right]t^{2}}{\Gamma \left(\gamma \right)\left(1-\mu^{2}\xi \right)\left[a\Gamma \left(\eta +3\right)\left(1-\xi \mu^{2}\right)+2b\Gamma \left(\eta +4\right)\left(1-\xi \mu^{3}\right)\right]}\int_{0}^{1}{\left(1-s\right)}^{\gamma -1}\\ & & L\left(s\right)ds-\frac{\Gamma \left(\eta +4\right)\Gamma \left(\eta +3\right)\left(1-\mu^{2}\xi \right)\left(1+t-\mu^{3}\xi \right)t^{2}}{\Gamma \left(\gamma -1\right)[a\Gamma \left(\eta +3\right)\left(1-\xi \mu^{2}\right)+2b\left(1-\xi \mu^{3}\right)\Gamma \left(\eta +4\right)]}\int_{0}^{1}{\left(1-s\right)}^{\gamma -2}L\left(s\right)ds\\ & & +\frac{\lambda \Gamma \left(\eta +3\right)\Gamma \left(\eta +4\right)\left(1-\xi \mu^{2}\right)\left(t^{2}\left(1-\xi \mu^{3}\right)+t^{3}\right)}{\Gamma \left(\gamma +\eta \right)[a\Gamma \left(\eta +3\right)\left(1-\xi \mu^{2}\right)+2b\left(1-\xi \mu^{3}\right)\Gamma \left(\eta +4\right)]}\int_{0}^{\nu }{\left(1-s\right)}^{\gamma +\eta -1}L\left(s\right)ds+\\ & & \frac{\xi t^{2}\left[a\Gamma \left(\eta +3\right)\left(1-\xi \mu^{2}\right)+2b\Gamma \left(\eta +4\right)\{\left(2-\xi \mu^{2}\right)\left(1-\xi \mu^{3}\right)+t\left(1-\xi \mu^{2}\right)\}\right]}{\Gamma \left(\gamma \right)\left(1-\xi \mu^{2}\right)\left[a\Gamma \left(\eta +3\right)\left(1-\xi \mu^{2}\right)+2b\Gamma \left(\eta +4\right)\left(1-\xi \mu^{3}\right)\right]}\int_{0}^{\mu }{\left(\mu -s\right)}^{\gamma -1}\\ & & L(s)ds. \end{array}$$ (12)

## 4 Existence and uniqueness

Some important notations are as follows:

$$\begin{array}{ccc} P_{1} & = & |\frac{1}{\Gamma (\gamma +1)}|+|\frac{t^{2}}{\left(1-\mu^{2}\xi \right)\Gamma \left(\gamma +1\right)}\\ & & \left\{\frac{\left[a\left(1-\mu^{2}\xi \right)\Gamma \left(\eta +3\right)+2b\left\{\left(2-\xi \mu^{2}\right)\left(1-\xi \mu^{3}\right)+t\left(1-\xi \mu^{2}\right)\right\}\Gamma \left(\eta +4\right)\right]}{\left[a\Gamma \left(\eta +3\right)\left(1-\xi \mu^{2}\right)+2b\Gamma \left(\eta +4\right)\left(1-\xi \mu^{3}\right)\right]}\right\}|\\ & & +|\frac{t^{2}\Gamma \left(\eta +3\right)\Gamma \left(\eta +4\right)\left(1-\xi \mu^{2}\right)(1+t-\mu^{3}\xi }{\Gamma \left(\gamma \right)[a\left(1-\xi \mu^{2}\right)\Gamma \left(\eta +3\right)+2b\left(1-\xi \mu^{3}\right)\Gamma \left(\eta +4\right)]}|\\ & & +|\frac{\nu^{\gamma +\eta }\lambda \Gamma \left(\eta +3\right)\Gamma \left(\eta +4\right)\left(1-\xi \mu^{2}\right)\left(t^{2}\left(1-\xi \mu^{3}\right)+t^{3}\right)}{\Gamma \left(\gamma +\eta +1\right)[a\Gamma \left(\eta +3\right)\left(1-\xi \mu^{2}\right)+2b\Gamma \left(\eta +4\right)\left(1-\xi \mu^{3}\right)]}|\\ & & +|\frac{\mu^{\gamma }\xi t^{2}\left[a\left(1-\xi \mu^{2}\right)\Gamma \left(\eta +3\right)+2b\left\{\left(2-\xi \mu^{2}\right)\left(1-\xi \mu^{3}\right)+t\left(1-\xi \mu^{2}\right)\right\}\Gamma \left(\eta +4\right)\right]}{\Gamma \left(\gamma +1\right)\left(1-\xi \mu^{2}\right)\left[a\Gamma \left(\eta +3\right)\left(1-\xi \mu^{2}\right)+2b\Gamma \left(\eta +4\right)\left(1-\xi \mu^{3}\right)\right]}|. \end{array}$$ (13)

$$\begin{array}{ccc} P_{2} & = & |\frac{1}{\Gamma (\gamma -\zeta +1)}|+|\frac{1}{\Gamma \left(\gamma +1\right)\left(1-\xi \mu^{2}\right)}\\ & & \frac{1}{a\Gamma \left(\eta +3\right)\left(1-\xi \mu^{2}\right)+2b\Gamma \left(\eta +4\right)\left(1-\xi \mu^{3}\right)}\{\frac{t^{2-\zeta }\left[a\Gamma \left(\eta +3\right)\left(1-\xi \mu^{2}\right)\right]}{\Gamma (3-\zeta )}\\ & & +\frac{2b\left(1-\xi \mu^{3}\right)\left(2-\xi \mu^{2}\right)\Gamma \left(\eta +4\right)t^{2-\zeta }}{\Gamma (3-\zeta )}+\frac{2bt^{3-\zeta }\left(1-\xi \mu^{2}\right)\Gamma \left(\eta +4\right)}{\Gamma (4-\zeta )}\}|\\ & & +|\frac{\left(1-\xi \mu^{2}\right)\Gamma \left(\eta +3\right)\Gamma \left(\eta +4\right)}{\Gamma \left(\gamma \right)[a\left(1-\xi \mu^{2}\right)\Gamma \left(\eta +3\right)+2b\left(1-\xi \mu^{3}\right)\Gamma \left(\eta +4\right)]}\{\frac{\left(1-\xi \mu^{3}\right)t^{2-\zeta }}{\Gamma (3-\zeta )}+\frac{t^{3-\zeta }}{\Gamma (4-\zeta )}\}|\\ & & +|\frac{\nu^{\gamma +\eta }\lambda \left(1-\xi \mu^{2}\right)\Gamma \left(\eta +3\right)\Gamma \left(\eta +4\right)}{\Gamma \left(\eta +\gamma +1\right)[a\Gamma \left(\eta +3\right)\left(1-\xi \mu^{2}\right)+2b\left(1-\xi \mu^{3}\right)\Gamma \left(\eta +4\right)]}\{\frac{t^{2-\zeta }\left(1-\xi \mu^{3}\right)}{\Gamma (3-\zeta )}\\ & & +\frac{t^{3-\zeta }}{\Gamma (4-\zeta )}\}|+|\frac{\xi \mu^{\gamma }}{\left(1-\xi \mu^{2}\right)\Gamma \left(\gamma +1\right)\left[a\left(1-\xi \mu^{2}\right)\Gamma \left(\eta +3\right)+2b\left(1-\xi \mu^{3}\right)\Gamma \left(\eta +4\right)\right]}\\ & & \{\frac{t^{2-\zeta }\left[a\left(1-\xi \mu^{2}\right)\Gamma \left(\eta +3\right)+2b\left(2-\xi \mu^{2}\right)\left(1-\xi \mu^{3}\right)\Gamma \left(\eta +4\right)\right]}{\Gamma (3-\zeta )}\\ & & +\frac{2b\left(1-\xi \mu^{2}\right)\Gamma \left(\eta +4\right)t^{3-\zeta }}{\Gamma (4-\zeta )}\}|. \end{array}$$ (14)

List of hypothesis for this paper are as follows:

(H1): Suppose $g:I\;R^{2}\to I\;R$ is a continuous function.

(H2): Suppose that there exists non negative real numbers *θ*, *ϑ* > 0 such that for all $(u,v),(u_{1},v_{1})\in I\;R^{2}$, we have

$$\begin{array}{c} |g\left(t,u,v\right)-g\left(t,u_{1},v_{1}\right)|<\theta \|u-u_{1}\|+\vartheta \|v-v_{1}\|. \end{array}$$ (15)

(H3): Suppose that a positive real number M exists such that |*g*(*t*, *u*, *v*)| ≤ *M* for all *u*, *v* ∈ *X* and for each *t* ∈ [0, 1].

**Theorem 4.1** Suppose that hypothesis (H2) holds and assume that 6λ*ν*^*η*+3^ ≠ Γ(*η* + 4) or λ*ν*^*η*+2^ ≠ Γ(*η* + 3).

If,

$$\begin{array}{c} \left(P_{1}+P_{2}\right)\left(\theta +\vartheta \right)<1, \end{array}$$ (16)

then in the interval [0, 1] the solution of problem (1) is unique.

**Proof** Suppose an operator *τ*: *X* → *X*, defined as:

*τu*(*t*) =

$$\begin{array}{ccc} & & \frac{1}{\Gamma (\gamma )}\int_{0}^{t}{\left(t-s\right)}^{\gamma -1}g\left(u\left(s\right),C^{\zeta }u\left(s\right)\right)ds-\\ & & \frac{\left[a\left(\left(1-\xi \mu^{3}\right)\Gamma \left(\eta +3\right)-\mu^{2}\xi \right)+2b\{\left(1-\xi \mu^{3}\right)\left(2-\mu^{2}\xi \right)+\left(1-\xi \mu^{2}\right)t\}\Gamma \left(\eta +4\right)\right]t^{2}}{\left(1-\mu^{2}\xi \right)\Gamma \left(\gamma \right)\left[a\left(1-\xi \mu^{2}\right)\Gamma \left(\eta +3\right)+2b\left(1-\xi \mu^{3}\right)\Gamma \left(\eta +4\right)\right]}\int_{0}^{1}{\left(1-s\right)}^{\gamma -1}\\ & & g\left(u\left(s\right),C^{\zeta }u\left(s\right)\right)ds-\frac{\Gamma \left(\eta +3\right)\Gamma \left(\eta +4\right)\left(1+t-\mu^{3}\xi \right)\left(1-\mu^{2}\xi \right)t^{2}}{\Gamma \left(\gamma -1\right)[a\left(1-\xi \mu^{2}\right)\Gamma \left(\eta +3\right)+2b\left(1-\xi \mu^{3}\right)\Gamma \left(\eta +4\right)]}\int_{0}^{1}{\left(1-s\right)}^{\gamma -2}\\ & & g\left(u\left(s\right),C^{\zeta }u\left(s\right)\right)ds+\frac{\lambda \Gamma \left(\eta +4\right)\Gamma \left(\eta +3\right)\left(t^{2}\left(1-\xi \mu^{3}\right)+t^{3}\right)\left(1-\xi \mu^{2}\right)}{\Gamma \left(\gamma +\eta \right)[a\left(1-\xi \mu^{2}\right)\Gamma \left(\eta +3\right)+2b\Gamma \left(\eta +4\right)\left(1-\xi \mu^{3}\right)]}\int_{0}^{\nu }{\left(1-s\right)}^{\gamma +\eta -1}\\ & & g\left(u\left(s\right),C^{\zeta }u\left(s\right)\right)ds+\frac{\xi t^{2}\left[a\left(1-\xi \mu^{2}\right)\Gamma \left(\eta +3\right)+2b\Gamma \left(\eta +4\right)\{\left(1-\xi \mu^{3}\right)\left(2-\xi \mu^{2}\right)+t\left(1-\xi \mu^{2}\right)\}\right]}{\left(1-\xi \mu^{2}\right)\Gamma \left(\gamma \right)\left[a\left(1-\xi \mu^{2}\right)\Gamma \left(\eta +3\right)+2b\left(1-\xi \mu^{3}\right)\Gamma \left(\eta +4\right)\right]}\\ & & \int_{0}^{\mu }{\left(\mu -s\right)}^{\gamma -1}g\left(u\left(s\right),C^{\zeta }u\left(s\right)\right)ds. \end{array}$$ (17)

For *u*, *v* ∈ *X* and for each *t* ∈ [0, 1], leads to

$$\begin{array}{ccc} \left|\tau u(t)-\tau v(t)\right| & \leq & |\frac{1}{\Gamma (\gamma )}|\int_{0}^{t}{\left(t-s\right)}^{\gamma -1}\left|g\left(u\left(s\right),C^{\zeta }u\left(s\right)\right)-g\left(v\left(s\right),C^{\zeta }v\left(s\right)\right)\right|ds+|\frac{t^{2}}{\Gamma (\gamma )}\\ & & \left\{\frac{a\left(1-\xi \mu^{2}\right)\Gamma \left(\eta +3\right)+2b[\left(2-\xi \mu^{2}\right)\left(1-\xi \mu^{3}\right)+t\left(1-\xi \mu^{2}\right)]\Gamma \left(\eta +4\right)}{\left(1-\xi \mu^{2}\right)\left[a\Gamma \left(\eta +3\right)\left(1-\xi \mu^{2}\right)+2b\Gamma \left(\eta +4\right)\left(1-\xi \mu^{3}\right)\right]}\}\right|\\ & & \int_{0}^{1}{\left(1-s\right)}^{\gamma -1}\left|g\left(u\left(s\right),C^{\zeta }u\left(s\right)\right)-g\left(v\left(s\right),C^{\zeta }v\left(s\right)\right)\right|ds\\ & & +|\frac{\Gamma \left(\eta +4\right)\Gamma \left(\eta +3\right)\left(1-\xi \mu^{2}\right)\left(1-\xi \mu^{3}+t\right)t^{2}}{\Gamma \left(\gamma -1\right)[a\left(1-\xi \mu^{2}\right)\Gamma \left(\eta +3\right)+2b\left(1-\xi \mu^{3}\right)\Gamma \left(\eta +4\right)]}|\\ & & \int_{0}^{1}{\left(1-s\right)}^{\gamma -2}\left|g\left(u\left(s\right),C^{\zeta }u\left(s\right)\right)-g\left(v\left(s\right),C^{\zeta }v\left(s\right)\right)\right|ds\\ & & +|\frac{\lambda \left(1-\xi \mu^{2}\right)\Gamma \left(\eta +4\right)\Gamma \left(\eta +3\right)\left(\left(1-\xi \mu^{3}\right)t^{2}\right)+t^{3})}{\Gamma \left(\eta +\gamma \right)[a\left(1-\xi \mu^{2}\right)\Gamma \left(\eta +3\right)+2b\left(1-\xi \mu^{3}\right)\Gamma \left(\eta +4\right)]}|\\ & & \int_{0}^{\nu }{\left(1-s\right)}^{\gamma +\eta -1}\left|g\left(u\left(s\right),C^{\zeta }u\left(s\right)\right)-g\left(v\left(s\right),C^{\zeta }v\left(s\right)\right)\right|ds\\ & & +|\frac{\xi t^{2}\{a\left(1-\xi \mu^{2}\Gamma \left(\eta +3\right)\right)+2b\Gamma \left(\eta +4\right)\left[\left(2-\xi \mu^{2}\right)\left(1-\xi \mu^{3}\right)+t\left(1-\xi \mu^{2}\right)\right]\}}{\left(1-\xi \mu^{2}\right)\Gamma \left(\gamma \right)\left[a\left(1-\xi \mu^{2}\right)\Gamma \left(\eta +3\right)+2b\left(1-\xi \mu^{3}\right)\Gamma \left(\eta +4\right)\right]}|\\ & & \int_{0}^{\mu }{\left(\mu -s\right)}^{\gamma -1}\left|g\left(u\left(s\right),C^{\zeta }u\left(s\right)\right)-g\left(v\left(s\right),C^{\zeta }v\left(s\right)\right)\right|ds. \end{array}$$ (18)

By (H2), we have

$$\begin{array}{ccc} \left|\tau u(t)-\tau v(t)\right| & \leq & \frac{\theta \left\|u-v\right\|+\vartheta \|C^{\zeta }u-C^{\zeta }v\|}{\left|\Gamma \left(\gamma +1\right)\right|}+\frac{\left(\theta \|u-v\|+\vartheta \|C^{\zeta }u-C^{\zeta }v\|\right)}{\left|\left(1-\xi \mu^{2}\right)\Gamma \left(\gamma +1\right)\right|}\\ & & \left\{\frac{|a\left(1-\xi \mu^{2}\right)\Gamma \left(\eta +3\right)+2b\left(1-\xi \mu^{3}\right)\Gamma \left(\eta +4\right)\left(3+\xi \mu^{2}\right)|}{|a\left(1-\xi \mu^{2}\right)\Gamma \left(\eta +3\right)+2b\left(1-\xi \mu^{3}\right)\Gamma \left(\eta +4\right)|}\right\}\\ & & +\frac{\left(\theta \|u-v\|+\vartheta \right\|C^{\zeta }u-C^{\zeta }v\|)|\Gamma \left(\eta +4\right)\Gamma \left(\eta +3\right)\left(2-\xi \mu^{3}\right)\left(1-\xi \mu^{2}\right)|}{\left|\Gamma \left(\gamma \right)[a\left(1-\xi \mu^{2}\right)\Gamma \left(\eta +3\right)+2b\left(1-\xi \mu^{3}\right)\Gamma \left(\eta +4\right)]\right|}\\ & & +|\frac{\lambda \nu^{\eta +\gamma }\Gamma \left(\eta +4\right)\Gamma \left(\eta +3\right)\left(2-\xi \mu^{3}\right)\left(1-\xi \mu^{2}\right)}{\Gamma \left(\eta +\gamma +1\right)[a\left(1-\xi \mu^{2}\right)\Gamma \left(\eta +3\right)+2b\left(1-\xi \mu^{3}\right)\Gamma \left(\eta +4\right)]}|\\ & & \left(\theta \|u-v\|+\vartheta \right\|C^{\zeta }u-C^{\zeta }v\left\|\right)+|\frac{\xi \mu^{\gamma }}{\left(1-\xi \mu^{2}\right)\Gamma \left(\gamma +1\right)}\\ & & \frac{\left[a\left(1-\xi \mu^{2}\right)\Gamma \left(\eta +3\right)+2b\left(1-\xi \mu^{3}\right)\Gamma \left(\eta +4\right)\left(3-\xi \mu^{2}\right)\right]}{\left[a\left(1-\xi \mu^{2}\right)\Gamma \left(\eta +3\right)+2b\left(1-\xi \mu^{3}\right)\Gamma \left(\eta +4\right)\right]}|\\ & & (\theta \|u-v\|+\vartheta \|C^{\zeta }u-C^{\zeta }v\|). \end{array}$$ (19)

Consequently we get,

$$\begin{array}{ccc} \left\|\tau u(t)-\tau v(t)\right\| & \leq & P_{1}\left(\theta +\vartheta \right)(\|u-v\|+\|C^{\zeta }u-C^{\zeta }v\left\|\right) \end{array}$$ (20)

and,

$$\begin{array}{ccc} |C^{\zeta }\tau u\left(t\right)-C^{\zeta }\tau v\left(t\right)| & \leq & |\frac{1}{\Gamma (\gamma -\zeta )}|\int_{0}^{t}{\left(t-s\right)}^{\gamma -\zeta -1}\left|g\left(u\left(s\right),C^{\zeta }u\left(s\right)\right)-g\left(v\left(s\right),C^{\zeta }v\left(s\right)\right)\right|ds\\ & & +|\frac{1}{\Gamma \left(\gamma \right)[a\left(1-\xi \mu^{2}\right)\Gamma \left(\eta +3\right)+2b\left(1-\xi \mu^{3}\right)\Gamma \left(\eta +4\right)]}\\ & & \{\frac{a\Gamma \left(\eta +3\right)t^{2-\zeta }}{\Gamma (3-\zeta )}+\frac{2bt^{3-\zeta }\Gamma \left(\eta +4\right)}{\Gamma (4-\zeta )}\\ & & +\frac{t^{2-\zeta }2b\left(2-\xi \mu^{2}\right)\Gamma \left(\eta +4\right)\left(1-\xi \mu^{3}\right)}{\left(1-\xi \mu^{2}\right)\Gamma \left(3-\zeta \right)}\}|\\ & & \int_{0}^{1}{\left(1-s\right)}^{\gamma -1}\left|g\left(u\left(s\right),C^{\zeta }u\left(s\right)\right)-g\left(v\left(s\right),C^{\zeta }v\left(s\right)\right)\right|ds\\ & & +|\frac{\left(1-\xi \mu^{2}\right)\Gamma \left(\eta +3\right)\Gamma \left(\eta +4\right)}{\Gamma \left(\gamma -1\right)[a\left(1-\xi \mu^{2}\right)\Gamma \left(\eta +3\right)+2b\left(1-\xi \mu^{3}\right)\Gamma \left(\eta +4\right)]}\\ & & \left\{\frac{t^{2-\zeta }\left(1-\xi \mu^{3}\right)}{\Gamma (3-\zeta )}+\frac{t^{3-\zeta }}{\Gamma (4-\zeta |)}\}\right|\\ & & \int_{0}^{1}{\left(1-s\right)}^{\gamma -2}\left|g\left(u\left(s\right),C^{\zeta }u\left(s\right)\right)-g\left(v\left(s\right),C^{\zeta }v\left(s\right)\right)\right|ds\\ & & +|\frac{\lambda \left(1-\xi \mu^{2}\right)\Gamma \left(\eta +3\right)\Gamma \left(\eta +4\right)}{\Gamma \left(\eta +\gamma \right)[a\left(1-\xi \mu^{2}\right)\Gamma \left(\eta +3\right)+2b\left(1-\xi \mu^{3}\right)\Gamma \left(\eta +4\right)]}\\ & & \left\{\frac{t^{2-\zeta }\left(1-\xi \mu^{3}\right)}{\Gamma (3-\zeta )}+\frac{t^{3-\zeta }}{\Gamma (4-\zeta )}\}\right|\\ & & \int_{0}^{\nu }{\left(\nu -s\right)}^{\gamma +\eta -1}\left|g\left(u\left(s\right),C^{\zeta }u\left(s\right)\right)-g\left(v\left(s\right),C^{\zeta }v\left(s\right)\right)\right|ds\\ & & +|\frac{\xi }{a\left(1-\xi \mu^{2}\right)\Gamma \left(\eta +3\right)+2b\left(1-\xi \mu^{3}\right)\Gamma \left(\eta +4\right)}\\ & & \{\frac{t^{2-\zeta }\left[a\left(1-\xi \mu^{2}\right)\Gamma \left(\eta +3\right)+2b\left(2-\xi \mu^{2}\right)\left(1-\xi \mu^{3}\right)\Gamma \left(\eta +4\right)\right]}{\Gamma \left(\gamma \right)\left(1-\xi \mu^{2}\right)\Gamma \left(3-\zeta \right)}\\ & & +\frac{2b\left(1-\xi \mu^{2}\right)\Gamma \left(\eta +4\right)t^{3-\zeta }}{\left(1-\xi \mu^{2}\right)\Gamma \left(\gamma \right)\Gamma \left(4-\zeta \right)}\}|\\ & & \int_{0}^{\mu }{\left(\mu -s\right)}^{\gamma -1}\left|g\left(u\left(s\right),C^{\zeta }u\left(s\right)\right)-g\left(v\left(s\right),C^{\zeta }v\left(s\right)\right)\right|. \end{array}$$ (21)

By hypothesis (H2), we have

$$\begin{array}{ccc} |C^{\zeta }\tau u\left(t\right)-C^{\zeta }\tau v\left(t\right)| & \leq & |\frac{1}{\Gamma (\zeta -\gamma +1)}|\left(\theta \|u-v\|+\vartheta \right\|C^{\zeta }u-C^{\zeta }v\left\|\right)\\ & & +|\frac{1}{\left[a\left(1-\xi \mu^{2}\right)\Gamma \left(\eta +3\right)+2b\left(1-\xi \mu^{3}\right)\Gamma \left(\eta +4\right)\right]}\\ & & \{\frac{t^{2-\zeta }\left[a\left(1-\xi \mu^{2}\right)\Gamma \left(\eta +3\right)+2b\left(2-\xi \mu^{2}\right)\Gamma \left(\eta +4\right)\left(1-\xi \mu^{3}\right)\right]}{\left(1-\xi \mu^{2}\right)\Gamma \left(\gamma +1\right)\Gamma \left(3-\zeta \right)}\\ & & +\frac{t^{3-\zeta }2b\left(1-\xi \mu^{2}\right)\Gamma \left(\eta +4\right)}{\Gamma (4-\zeta )\Gamma (\gamma +1)}\}|\left(\theta \|u-v\|+\vartheta \right\|C^{\zeta }u-C^{\zeta }v\left\|\right)\\ & & +|\frac{\left(1-\xi \mu^{2}\right)\Gamma \left(\eta +3\right)\Gamma \left(\eta +4\right)}{\Gamma \left(\gamma \right)[a\left(1-\xi \mu^{2}\right)\Gamma \left(\eta +3\right)+2b\left(1-\xi \mu^{3}\right)\Gamma \left(\eta +4\right)]}\\ & & \{\frac{\left(1-\xi \mu^{3}\right)}{\Gamma (3-\zeta )}+\frac{t^{3-\zeta }}{\Gamma (4-\zeta |)}\}|\left(\theta \|u-v\|+\vartheta \right\|C^{\zeta }u-C^{\zeta }v\left\|\right)\\ & & +|\frac{\lambda \nu^{\gamma +\eta }\left(1-\xi \mu^{2}\right)\Gamma \left(\eta +3\right)\Gamma \left(\eta +4\right)}{\Gamma \left(\gamma +\eta +1\right)[a\left(1-\xi \mu^{2}\right)\Gamma \left(\eta +3\right)+2b\left(1-\xi \mu^{3}\right)\Gamma \left(\eta +4\right)]}\\ & & \{\frac{t^{2-\zeta }\left(1-\xi \mu^{3}\right)}{\Gamma (3-\zeta )}+\frac{t^{3-\zeta }}{\Gamma (4-\zeta )}\}|\left(\theta \|u-v\|+\vartheta \right\|C^{\zeta }u-C^{\zeta }v\left\|\right)\\ & & +|\frac{\xi \mu^{\gamma }}{\left(1-\xi \mu^{2}\right)\Gamma \left(\gamma +1\right)\left[a\left(1-\xi \mu^{2}\right)\Gamma \left(\eta +3\right)+2b\left(1-\xi \mu^{3}\right)\Gamma \left(\eta +4\right)\right]}\\ & & \{\frac{t^{2-\zeta }\left[a\left(1-\xi \mu^{2}\right)\Gamma \left(\eta +3\right)+2b\left(2-\xi \mu^{2}\right)\Gamma \left(\eta +4\right)\left(1-\xi \mu^{3}\right)\right]}{\Gamma (3-\zeta )}\\ & & +\frac{2b\left(1-\xi \mu^{2}\right)\Gamma \left(\eta +4\right)t^{3-\zeta }}{\Gamma (4-\zeta )}\}|\left(\theta \|u-v\|+\vartheta \right\|C^{\zeta }u-C^{\zeta }v\left\|\right), \end{array}$$ (22)

which implies that,

$$\begin{array}{ccc} & \|C^{\zeta }\tau u\left(t\right)-C^{\zeta }\tau v\left(t\right)\| & \leq P_{2}\left(\theta +\vartheta \right)(\|u-v\|+\|C^{\zeta }u-C^{\zeta }v\left\|\right). \end{array}$$ (23)

From Eqs (18) and (22), it can be written as:

$$\begin{array}{c} {\left\|\tau \left(u\right)-\tau \left(v\right)\right\|}_{X}\leq \left(P_{1}+P_{2}\right)\left(\theta +\vartheta \right)\left(\|u-v\|+\right\|C^{\zeta }u-C^{\zeta }v\left\|\right). \end{array}$$ (24)

We deduce that *τ* is a contraction. As consequence of Banach Fixed contraction principle, the problem (1) has a unique solution.

**Theorem 4.2** Assume that the (H1) and (H3) hold and suppose that 6λ*ν*^*η*+3^ ≠ Γ(*η* + 4) or λ*ν*^*η*+2^ ≠ Γ(*η* + 3). Then atleast one solution of BVP (1) exists in interval [0, 1].

**Proof**: The proof of this theorem will be given in four steps.

**Step 1**: In *X* the operator *τ* is continuous.

It is obvious that the operator *τ* is continuous because the function *g* is continuous function.

**Step 2**: The operator *τ* maps bounded sets into bounded sets in *X*:

For *ω* > 0 we take *B*_*ω*_ = {*u* ∈ *B*; ‖*u*‖_*X*_ ≤ *ω*}.

For *u* ∈ *B*_*ω*_ and *t* ∈ [0, 1], implies

$$\begin{array}{ccc} \left|\tau u(t)\right| & = & |\frac{1}{\Gamma (\gamma )}|\int_{0}^{t}{\left(t-s\right)}^{\gamma -1}\left|g\left(u\left(s\right),C^{\zeta }u\left(s\right)\right)\right|ds\\ & & +|\frac{t^{2}}{\left[a\left(1-\xi \mu^{2}\right)\Gamma \left(\eta +3\right)+2b\left(1-\xi \mu^{3}\right)\Gamma \left(\eta +4\right)\right]}\{\frac{a\Gamma (\eta +3)}{\Gamma (\gamma )}+\frac{t}{\Gamma (\gamma )}\\ & & +\frac{2b\left(2-\xi \mu^{2}\right)\Gamma \left(\eta +4\right)\left(1-\xi \mu^{3}\right)}{\left(1-\xi \mu^{2}\right)\Gamma \left(\gamma \right)}\}|\int_{0}^{1}{\left(1-s\right)}^{\gamma -1}\left|g\left(u\left(s\right),C^{\zeta }u\left(s\right)\right)\right|ds\\ & & +|\frac{t^{2}\Gamma \left(\eta +4\right)\Gamma \left(\eta +3\right)\left(1-\xi \mu^{3}+t\right)\left(1-\xi \mu^{2}\right)}{\Gamma \left(\gamma -1\right)[a\left(1-\xi \mu^{2}\right)\Gamma \left(\eta +3\right)+2b\left(1-\xi \mu^{3}\right)\Gamma \left(\eta +4\right)]}|\\ & & \int_{0}^{1}{\left(1-s\right)}^{\gamma -2}\left|g\left(u\left(s\right),C^{\zeta }u\left(s\right)\right)\right|ds+|\frac{\lambda }{\Gamma (\eta +\gamma )}\\ & & \frac{\Gamma \left(\eta +4\right)\left(1-\xi \mu^{2}\right)\Gamma \left(\eta +3\right)\left(t^{2}\left(1-\xi \mu^{3}\right)+t^{3}\right)}{\left[a\left(1-\xi \mu^{2}\right)\Gamma \left(\eta +3\right)+2b\left(1-\xi \mu^{3}\right)\Gamma \left(\eta +4\right)\right]}|\int_{0}^{\nu }{\left(1-s\right)}^{\gamma +\eta -1}\left|g\left(u\left(s\right),C^{\zeta }u\left(s\right)\right)\right|ds\\ & & +|\frac{\xi t^{2}\left[a\left(1-\xi \mu^{2}\right)\Gamma \left(\eta +3\right)+2b\left[\left(2-\xi \mu^{2}\right)\Gamma \left(\eta +4\right)\left(1-\xi \mu^{3}\right)+t\left(1-\xi \mu^{2}\right)\right]\right]}{\Gamma \left(\gamma \right)\left(1-\xi \mu^{2}\right)\left[a\left(1-\xi \mu^{2}\right)\Gamma \left(\eta +3\right)+2b\left(1-\xi \mu^{3}\right)\Gamma \left(\eta +4\right)\right]}|\\ & & \int_{0}^{\mu }{\left(\mu -s\right)}^{\gamma -1}\left|g\left(u\left(s\right),C^{\zeta }u\left(s\right)\right)\right|ds. \end{array}$$ (25)

By (H3), the above relation can be express as

$$\begin{array}{ccc} \left|\tau u(t)\right| & \leq & |\frac{1}{\Gamma (\gamma +1)}|M+|\frac{t^{2}}{\left(1-\xi \mu^{2}\right)\Gamma \left(\gamma +1\right)}\\ & & \{\frac{a\left(1-\xi \mu^{2}\right)\Gamma \left(\eta +3\right)+2b\left[\left(1-\xi \mu^{3}\right)\left(2-\xi \mu^{2}\right)+\left(1-\xi \mu^{2}\right)t\right]\Gamma \left(\eta +4\right)}{\left[a\left(1-\xi \mu^{2}\right)\Gamma \left(\eta +3\right)+2b\left(1-\xi \mu^{3}\right)\Gamma \left(\eta +4\right)\right]}\}|M\\ & & +|\frac{t^{2}\Gamma \left(\eta +4\right)\left(1-\xi \mu^{2}\right)\Gamma \left(\eta +3\right)\left(1-\xi \mu^{3}+t\right)}{\Gamma \left(\gamma \right)[a\left(1-\xi \mu^{2}\right)\Gamma \left(\eta +3\right)+2b\left(1-\xi \mu^{3}\right)\Gamma \left(\eta +4\right)]}|M\\ & & +|\frac{\lambda \nu^{\eta +\gamma }\Gamma \left(\eta +3\right)\left(1-\xi \mu^{2}\right)\Gamma \left(\eta +4\right)\left(t^{2}\left(1-\xi \mu^{3}\right)+t^{3}\right)}{\Gamma \left(\gamma +\eta +1\right)[a\left(1-\xi \mu^{2}\right)\Gamma \left(\eta +3\right)+2b\left(1-\xi \mu^{3}\right)\Gamma \left(\eta +4\right)]}|M\\ & & +|\frac{\xi \mu^{\gamma }t^{2}\left\{a\left(1-\xi \mu^{2}\right)\Gamma \left(\eta +3\right)+2b\left[\left(2-\xi \mu^{2}\right)\Gamma \left(\eta +4\right)\left(1-\xi \mu^{3}\right)+\left(1-\xi \mu^{2}\right)t\right]\right\}}{\Gamma \left(\gamma +1\right)\left(1-\xi \mu^{2}\right)\left[a\left(1-\xi \mu^{2}\right)\Gamma \left(\eta +3\right)+2b\left(1-\xi \mu^{3}\right)\Gamma \left(\eta +4\right)\right]}|M\\ & = & M[|\frac{1}{\Gamma (\gamma +1)}|+|\frac{t^{2}}{\left(1-\mu^{2}\xi \right)\Gamma \left(\gamma +1\right)}\\ & & \left\{\frac{\left[a\left(1-\mu^{2}\xi \right)\Gamma \left(\eta +3\right)+2b\left\{\left(2-\xi \mu^{2}\right)\left(1-\xi \mu^{3}\right)+t\left(1-\xi \mu^{2}\right)\right\}\Gamma \left(\eta +4\right)\right]}{\left[a\Gamma \left(\eta +3\right)\left(1-\xi \mu^{2}\right)+2b\Gamma \left(\eta +4\right)\left(1-\xi \mu^{3}\right)\right]}\}\right|\\ & & +|\frac{t^{2}\Gamma \left(\eta +3\right)\Gamma \left(\eta +4\right)\left(1-\xi \mu^{2}\right)(1+t-\mu^{3}\xi }{\Gamma \left(\gamma \right)[a\left(1-\xi \mu^{2}\right)\Gamma \left(\eta +3\right)+2b\left(1-\xi \mu^{3}\right)\Gamma \left(\eta +4\right)]}|\\ & & +|\frac{\nu^{\gamma +\eta }\lambda \Gamma \left(\eta +3\right)\Gamma \left(\eta +4\right)\left(1-\xi \mu^{2}\right)\left(t^{2}\left(1-\xi \mu^{3}\right)+t^{3}\right)}{\Gamma \left(\gamma +\eta +1\right)[a\Gamma \left(\eta +3\right)\left(1-\xi \mu^{2}\right)+2b\Gamma \left(\eta +4\right)\left(1-\xi \mu^{3}\right)]}\\ & & +\frac{\mu^{\gamma }\xi t^{2}\left[a\left(1-\xi \mu^{2}\right)\Gamma \left(\eta +3\right)+2b\left\{\left(2-\xi \mu^{2}\right)\left(1-\xi \mu^{3}\right)+t\left(1-\xi \mu^{2}\right)\right\}\Gamma \left(\eta +4\right)\right]}{\Gamma \left(\gamma +1\right)\left(1-\xi \mu^{2}\right)\left[a\left(1-\xi \mu^{2}\right)\Gamma \left(\eta +3\right)+2b\left(1-\xi \mu^{3}\right)\Gamma \left(\eta +4\right)\right]}|]. \end{array}$$ (26)

which implies,

$$\begin{array}{ccc} \left|\tau u(t)\right| & \leq & MP_{1}. \end{array}$$ (27)

Therefore,

$$\begin{array}{c} \|\tau (t)\|<\infty . \end{array}$$ (28)

Consequently,

$$\begin{array}{ccc} |C^{\zeta }\tau u\left(t\right)| & \leq & |\frac{1}{\Gamma (\gamma -\zeta )}|\int_{0}^{t}{\left(t-s\right)}^{\gamma -\zeta -1}\left|g\left(u\left(s\right),C^{\zeta }u\left(s\right)\right)\right|ds\\ & & +|\frac{1}{\left(1-\xi \mu^{2}\right)\Gamma \left(\gamma \right)\{a\left(1-\xi \mu^{2}\right)\Gamma \left(\eta +3\right)+2b\left(1-\xi \mu^{3}\right)\Gamma \left(\eta +4\right)\}}\\ & & \{\frac{t^{2-\zeta }\left[a\left(1-\xi \mu^{2}\right)\Gamma \left(\eta +3\right)+2b\left(2-\xi \mu^{2}\right)\Gamma \left(\eta +4\right)\left(1-\xi \mu^{3}\right)\right]}{\Gamma (3-\zeta )}\\ & & +\frac{t^{3-\zeta }2b\left(1-\xi \mu^{2}\right)\Gamma \left(\eta +4\right)}{\Gamma (4-\zeta )}\}|\int_{0}^{1}{\left(1-s\right)}^{\gamma -1}\left|g\left(u\left(s\right),C^{\zeta }u\left(s\right)\right)\right|ds\\ & & +|\frac{\left(1-\xi \mu^{2}\right)\Gamma \left(\eta +3\right)\Gamma \left(\eta +4\right)}{\Gamma \left(\gamma -1\right)\left[a\right)\left(1-\xi \mu^{2}\right)\Gamma \left(\eta +3+2b\left(1-\xi \mu^{3}\right)\Gamma \left(\eta +4\right)\right]}\\ & & \{\frac{t^{3-\zeta }}{\Gamma (4-\zeta |)}+\frac{\left(1-\xi \mu^{3}\right)t^{2-\zeta }}{\Gamma (3-\zeta )}\}|\int_{0}^{1}{\left(1-s\right)}^{\gamma -2}\left|g\left(u\left(s\right),C^{\zeta }u\left(s\right)\right)\right|ds\\ & & +|\frac{\lambda \Gamma \left(\eta +3\right)\left(1-\xi \mu^{2}\right)\Gamma \left(\eta +4\right)}{\Gamma \left(\eta +\gamma \right)[a\left(1-\xi \mu^{2}\right)\Gamma \left(\eta +3\right)+2b\left(1-\xi \mu^{3}\right)\Gamma \left(\eta +4\right)]}\\ & & \{\frac{\left(1-\xi \mu^{3}\right)t^{2-\zeta }}{\Gamma (3-\zeta )}+\frac{t^{3-\zeta }}{\Gamma (4-\zeta )}\}|\int_{0}^{\nu }{\left(\nu -s\right)}^{\gamma +\eta -1}\left|g\left(u\left(s\right),C^{\zeta }u\left(s\right)\right)\right|ds\\ & & +|\frac{\mu^{\gamma }\xi }{\left(1-\xi \mu^{2}\right)\Gamma \left(\gamma \right)\left[a\Gamma \left(\eta +3\right)\left(1-\xi \mu^{2}\right)+2b\left(1-\xi \mu^{3}\right)\Gamma \left(\eta +4\right)\right]}\\ & & \{\frac{t^{2-\zeta }\left[a\left(1-\xi \mu^{2}\right)\Gamma \left(\eta +3\right)+2b\left(2-\xi \mu^{2}\right)\Gamma \left(\eta +4\right)\left(1-\xi \mu^{3}\right)\right]}{\Gamma (3-\zeta )}\\ & & +\frac{2b\left(1-\xi \mu^{2}\right)\Gamma \left(\eta +4\right)t^{3-\zeta }}{\Gamma (4-\zeta )}\}|\int_{0}^{\mu }{\left(\mu -s\right)}^{\gamma -1}\left|g\left(u\left(s\right),C^{\zeta }u\left(s\right)\right)\right|ds\\ & \leq & |\frac{1}{\Gamma (\gamma -\zeta +1)}|M+|\frac{1}{\left(1-\xi \mu^{2}\right)\Gamma \left(\gamma +1\right)}\\ & & \frac{1}{\left\{a\left(1-\xi \mu^{2}\right)\Gamma \left(\eta +3\right)+2b\left(1-\xi \mu^{3}\right)\Gamma \left(\eta +4\right)\right\}}\{\frac{t^{2-\zeta }a\left(1-\xi \mu^{2}\right)\Gamma \left(\eta +3\right)}{\Gamma (3-\zeta )}\\ & & +\frac{2b\left(2-\xi \mu^{2}\right)\Gamma \left(\eta +4\right)\left(1-\xi \mu^{3}\right)t^{2-\zeta }}{\Gamma (3-\zeta )}+\frac{t^{3-\zeta }2b\left(1-\xi \mu^{2}\right)\Gamma \left(\eta +4\right)}{\Gamma (4-\zeta )}\}|M\\ & & +|\frac{\left(1-\xi \mu^{2}\right)\Gamma \left(\eta +3\right)\Gamma \left(\eta +4\right)}{\Gamma \left(\gamma \right)[a\left(1-\xi \mu^{2}\right)\Gamma \left(\eta +3\right)+2b\left(1-\xi \mu^{3}\right)\Gamma \left(\eta +4\right)]}\{\frac{t^{2-\zeta }\left(1-\xi \mu^{3}\right)}{\Gamma (3-\zeta )}+\frac{t^{3-\zeta }}{\Gamma (4-\zeta |)}\}|M\\ & & +|\frac{\nu^{\eta +\gamma }\lambda \left(1-\xi \mu^{2}\right)\Gamma \left(\eta +4\right)\Gamma \left(\eta +3\right)}{\Gamma \left(\eta +\gamma +1\right)[a\left(1-\xi \mu^{2}\right)\Gamma \left(\eta +3\right)+2b\left(1-\xi \mu^{3}\right)\Gamma \left(\eta +4\right)]}\{\frac{t^{2-\zeta }\left(1-\xi \mu^{3}\right)}{\Gamma (3-\zeta )}\\ & & +\frac{t^{3-\zeta }}{\Gamma (4-\zeta )}\}|M+|\frac{\mu^{\gamma }\xi }{\Gamma \left(\gamma +1\right)\left(1-\xi \mu^{2}\right)\left[a\left(1-\xi \mu^{2}\right)\Gamma \left(\eta +3\right)+2b\left(1-\xi \mu^{3}\right)\Gamma \left(\eta +4\right)\right]}\\ & & \{\frac{t^{2-\zeta }\left[a\left(1-\xi \mu^{2}\right)\Gamma \left(\eta +3\right)+2b\left(2-\xi \mu^{2}\right)\Gamma \left(\eta +4\right)\left(1-\xi \mu^{3}\right)\right]}{\Gamma (3-\zeta )}\\ & & +\frac{2b\left(1-\xi \mu^{2}\right)\Gamma \left(\eta +4\right)t^{3-\zeta }}{\Gamma (4-\zeta )}\}|M\\ & = & M[|\frac{1}{\Gamma (\gamma -\zeta +1)}|+|\frac{1}{\Gamma \left(\gamma +1\right)\left(1-\xi \mu^{2}\right)\Gamma \left(\eta +4\right)}\\ & & \frac{1}{a\left(1-\xi \mu^{2}\right)\Gamma \left(\eta +3\right)+2b\left(1-\xi \mu^{3}\right)\Gamma \left(\eta +4\right)}\{\frac{t^{2-\zeta }\left[a\left(1-\xi \mu^{2}\right)\Gamma \left(\eta +3\right)\right]}{\Gamma (3-\zeta )}\\ & & +\frac{2b\left(2-\xi \mu^{2}\right)\Gamma \left(\eta +4\right)\left(1-\xi \mu^{3}\right)t^{2-\zeta }}{\Gamma (3-\zeta )}+\frac{t^{3-\zeta }2b\left(1-\xi \mu^{2}\right)\Gamma \left(\eta +4\right)}{\Gamma (4-\zeta )}\}|\\ & & +|\frac{\left(1-\xi \mu^{2}\right)\Gamma \left(\eta +3\right)\Gamma \left(\eta +4\right)}{\Gamma \left(\gamma \right)[a\left(1-\xi \mu^{2}\right)\Gamma \left(\eta +3\right)+2b\left(1-\xi \mu^{3}\right)\Gamma \left(\eta +4\right)]}\{\frac{t^{2-\zeta }\left(1-\xi \mu^{3}\right)}{\Gamma (3-\zeta )}+\frac{t^{3-\zeta }}{\Gamma (4-\zeta |)}\}|\\ & & +|\frac{\nu^{\gamma +\eta }\lambda \left(1-\xi \mu^{2}\right)\Gamma \left(\eta +3\right)\Gamma \left(\eta +4\right)}{\Gamma \left(\gamma +\eta +1\right)[a\left(1-\xi \mu^{2}\right)\Gamma \left(\eta +3\right)+2b\left(1-\xi \mu^{3}\right)\Gamma \left(\eta +4\right)]}\{\frac{t^{2-\zeta }\left(1-\xi \mu^{3}\right)}{\Gamma (3-\zeta )}\\ & & +\frac{t^{3-\zeta }}{\Gamma (4-\zeta )}\}|+|\frac{\mu^{\gamma }\xi }{\left(1-\xi \mu^{2}\right)\Gamma \left(\gamma +1\right)\left[a\left(1-\xi \mu^{2}\right)\Gamma \left(\eta +3\right)+2b\left(1-\xi \mu^{3}\right)\Gamma \left(\eta +4\right)\right]}\\ & & \{\frac{t^{2-\zeta }\left[a\left(1-\xi \mu^{2}\right)\Gamma \left(\eta +3\right)+2b\left(2-\xi \mu^{2}\right)\Gamma \left(\eta +4\right)\left(1-\xi \mu^{3}\right)\right]}{\Gamma (3-\zeta )}\\ & & +\frac{2b\left(1-\xi \mu^{2}\right)\Gamma \left(\eta +4\right)t^{3-\zeta }}{\Gamma (4-\zeta )}\}|]. \end{array}$$ (29)

The above expression can be written as:

$$\begin{array}{ccc} \|C^{\zeta }\tau u\left(t\right)\| & \leq & MP_{2}. \end{array}$$ (30)

Using Eqs 27 and 30 gives,

$$\begin{array}{c} {\left\|\tau \left(u\right)\right\|}_{X}\leq M\left(P_{1}+P_{2}\right), \end{array}$$ (31)

which implies,

$$\begin{array}{c} {\left\|\tau \left(u\right)\right\|}_{X}<\infty . \end{array}$$ (32)

**Step 3**: In this step we will prove that the operator *τ* is equi-continuous on *J*:

Assume that if *t*_1_, *t*_2_ where *t*_2_ > *t*_1_ and *u* ∈ *B*_*ω*_., then,

$$\begin{array}{ccc} |\tau u\left(t_{2}\right)-\tau u\left(t_{1}\right)| & \leq & |\frac{1}{\Gamma (\gamma )}|\int_{0}^{t_{1}}\left({\left(t_{2}-s\right)}^{\gamma -1}-{\left(t_{1}-s\right)}^{\gamma -1}\right)\left|g\left(u\left(s\right),C^{\zeta }u\left(s\right)\right)\right|ds\\ & & +|\frac{1}{\Gamma (\gamma )}|\int_{t_{1}}^{t_{2}}{\left(t_{2}-s\right)}^{\gamma -1}\left|g\left(u\left(s\right),C^{\zeta }u\left(s\right)\right)\right|ds+|\frac{1}{\Gamma \left(\gamma \right)\left(1-\xi \mu^{2}\right)\Gamma \left(\gamma \right)}\\ & & \{\frac{\left(t_{2}^{2}-t_{1}^{2}\right)\left[a\left(1-\xi \mu^{2}\right)\Gamma \left(\eta +3\right)+2b\left(2-\xi \mu^{2}\right)\Gamma \left(\eta +4\right)\left(1-\xi \mu^{3}\right)\right]}{a\left(1-\xi \mu^{2}\right)\Gamma \left(\eta +3\right)+2b\Gamma \left(\eta +4\right)\left(1-\xi \mu^{3}\right)\Gamma \left(\eta +4\right)}\\ & & +\frac{\left(t_{2}^{3}-t_{1}^{3}\right)2b\left(1-\xi \mu^{2}\right)\Gamma \left(\eta +4\right)}{a\Gamma \left(\eta +3\right)\left(1-\xi \mu^{2}\right)\Gamma \left(\eta +3\right)+2b\left(1-\xi \mu^{3}\right)\Gamma \left(\eta +4\right)}\}|\\ & & \int_{0}^{1}{\left(1-s\right)}^{\gamma -1}\left|g\left(u\left(s\right),C^{\zeta }u\left(s\right)\right)\right|ds\\ & & +|\{\frac{\left(1-\xi \mu^{2}\right)\Gamma \left(\eta +3\right)\left(1-\xi \mu^{3}\right)\Gamma \left(\eta +4\right)\left(t_{2}^{2}-t_{1}^{2}\right)}{\Gamma \left(\gamma -1\right)[a\left(1-\xi \mu^{2}\right)\Gamma \left(\eta +3\right)+2b\left(1-\xi \mu^{3}\right)\Gamma \left(\eta +4\right)]}\\ & & +\frac{\left(1-\xi \mu^{2}\right)\Gamma \left(\eta +3\right)\Gamma \left(\eta +4\right)\left(t_{2}^{3}-t_{1}^{3}\right)}{\Gamma \left(\gamma -1\right)[a\left(1-\xi \mu^{2}\right)\Gamma \left(\eta +3\right)+2b\left(1-\xi \mu^{3}\right)\Gamma \left(\eta +4\right)]}\}|\\ & & \int_{0}^{1}{\left(1-s\right)}^{\gamma -2}\left|g\left(u\left(s\right),C^{\zeta }u\left(s\right)\right)\right|ds\\ & & +|\{\frac{\lambda \left(1-\xi \mu^{2}\right)\Gamma \left(\eta +3\right)\left(1-\xi \mu^{3}\right)\Gamma \left(\eta +4\right)\left(t_{2}^{2}-t_{1}^{2}\right)}{\Gamma \left(\eta +\gamma \right)[a\left(1-\xi \mu^{2}\right)\Gamma \left(\eta +3\right)+2b\Gamma \left(\eta +4\right)\left(1-\xi \mu^{3}\right)\Gamma \left(\eta +4\right)]}\\ & & +\frac{\lambda \left(1-\xi \mu^{2}\right)\Gamma \left(\eta +3\right)\Gamma \left(\eta +4\right)\left(t_{2}^{3}-t_{1}^{3}\right)}{\Gamma \left(\eta +\gamma \right)[a\left(1-\xi \mu^{2}\right)\Gamma \left(\eta +3\right)+2b\left(1-\xi \mu^{3}\right)\Gamma \left(\eta +4\right)]}\}|\\ & & \int_{0}^{\nu }{\left(\nu -s\right)}^{\gamma +\eta -1}\left|g\left(u\left(s\right),C^{\zeta }u\left(s\right)\right)\right|ds+|\frac{\xi }{\left(1-\xi \mu^{2}\right)\Gamma \left(\gamma \right)}\\ & & \{\frac{\left[a\left(1-\xi \mu^{2}\right)\Gamma \left(\eta +3\right)+2b\left(2-\xi \mu^{2}\right)\Gamma \left(\eta +4\right)\left(1-\xi \mu^{3}\right)\right]\left(t_{2}^{2}-t_{1}^{2}\right)}{\left[a\left(1-\xi \mu^{2}\right)\Gamma \left(\eta +3\right)+2b\left(1-\xi \mu^{3}\right)\Gamma \left(\eta +4\right)\right]}\\ & & +\frac{2b\xi \left(1-\xi \mu^{2}\right)\Gamma \left(\eta +4\right)\left(t_{2}^{3}-t_{1}^{3}\right)}{\left[a\left(1-\xi \mu^{2}\right)\Gamma \left(\eta +3\right)+2b\left(1-\xi \mu^{3}\right)\Gamma \left(\eta +4\right)\right]}\}|\\ & & \int_{0}^{\mu }{\left(\mu -s\right)}^{\gamma -1}\left|g\left(u\left(s\right),C^{\zeta }u\left(s\right)\right)\right|ds. \end{array}$$ (33)

Using (H3),

$$\begin{array}{ccc} |\tau u\left(t_{2}\right)-\tau u\left(t_{1}\right)| & \leq & \frac{M}{\left|\Gamma (\gamma +1)\right|}\left(t_{1}^{\gamma }-t_{2}^{\gamma }\right)+\frac{M}{\left|\Gamma (\gamma +1)\right|}{\left(t_{2}-t_{1}\right)}^{\gamma }+\frac{M}{|\left(1-\xi \mu^{2}\right)\Gamma \left(\gamma +1\right)|}\\ & & |\{\frac{\left(t_{2}^{2}-t_{1}^{2}\right)\left[a\left(1-\xi \mu^{2}\right)\Gamma \left(\eta +3\right)+2b\Gamma \left(\left(2-\xi \mu^{2}\right)\eta +4\right)\left(1-\xi \mu^{3}\right)\right]}{a\left(1-\xi \mu^{2}\right)\Gamma \left(\eta +3\right)+2b\left(1-\xi \mu^{3}\right)\Gamma \left(\eta +4\right)}\\ & & +\frac{\left(t_{2}^{3}-t_{1}^{3}\right)2b\left(1-\xi \mu^{2}\right)\Gamma \left(\eta +4\right)}{a\left(1-\xi \mu^{2}\right)\Gamma \left(\eta +3\right)+2b\left(1-\xi \mu^{3}\right)\Gamma \left(\eta +4\right)}\}|\\ & & +\frac{M|\left(1-\xi \mu^{2}\right)\Gamma \left(\eta +3\right)\Gamma \left(\eta +4\right)\left(1-\xi \mu^{3}\right)\left(t_{2}^{2}-t_{1}^{2}\right)|}{\left|\Gamma \left(\gamma \right)[a\left(1-\xi \mu^{2}\right)\Gamma \left(\eta +3\right)+2b\left(1-\xi \mu^{3}\right)]\right|}\\ & & +\frac{M|\left(1-\xi \mu^{2}\right)\Gamma \left(\eta +4\right)\Gamma \left(\eta +3\right)\left(t_{2}^{3}-t_{1}^{3}\right)|}{\left|\Gamma \left(\gamma \right)[a\left(1-\xi \mu^{2}\right)\Gamma \left(\eta +3\right)+2b\left(1-\xi \mu^{3}\right)\Gamma \left(\eta +4\right)]\right|}\\ & & +\frac{M|\nu^{\gamma +\eta }\lambda \left(1-\xi \mu^{2}\right)\Gamma \left(\eta +3\right)\Gamma \left(\eta +4\right)\left(1-\xi \mu^{3}\right)\left(t_{2}^{2}-t_{1}^{2}\right)|}{\left|\Gamma \left(\gamma +\eta +1\right)[a\left(1-\xi \mu^{2}\right)\Gamma \left(\eta +3\right)+2b\left(1-\xi \mu^{3}\right)\Gamma \left(\eta +4\right)]\right|}\\ & & +\frac{M|\nu^{\eta +\gamma }\lambda \left(1-\xi \mu^{2}\right)\Gamma \left(\eta +3\right)\Gamma \left(\eta +4\right)\left(t_{2}^{3}-t_{1}^{3}\right)|}{\left|\Gamma \left(\eta +\gamma +1\right)[a\left(1-\xi \mu^{2}\right)\Gamma \left(\eta +3\right)+2b\left(1-\xi \mu^{3}\right)\Gamma \left(\eta +4\right)]\right|}\\ & & +\frac{M|\mu^{\gamma }\xi \left[a\left(1-\xi \mu^{2}\right)\Gamma \left(\eta +3\right)+2b\left(2-\xi \mu^{2}\right)\left(1-\xi \mu^{3}\right)\Gamma \left(\eta +4\right)\right]\left(t_{2}^{2}-t_{1}^{2}\right)|}{|\left(1-\xi \mu^{2}\right)\Gamma \left(\gamma +1\right)\left[a\left(1-\xi \mu^{2}\right)\Gamma \left(\eta +3\right)+2b\left(1-\xi \mu^{3}\right)\Gamma \left(\eta +4\right)\right]|}\\ & & +\frac{2M|\mu^{\gamma }b\xi \left(1-\xi \mu^{2}\right)\Gamma \left(\eta +4\right)\left(t_{2}^{3}-t_{1}^{3}\right)|}{|\left(1-\xi \mu^{2}\right)\Gamma \left(\gamma +1\right)\left[a\left(1-\xi \mu^{2}\right)\Gamma \left(\eta +3\right)+2b\left(1-\xi \mu^{3}\right)\Gamma \left(\eta +4\right)\right]|} \end{array}$$ (34)

and,

$$\begin{array}{ccc} |C^{\zeta }\tau u\left(t_{2}\right)-C^{\zeta }\tau u\left(t_{1}\right)| & \leq & |\frac{1}{\Gamma (\gamma -\zeta )}|\int_{0}^{t_{1}}\left({\left(t_{1}-s\right)}^{\gamma -\zeta -1}-{\left(t_{2}-s\right)}^{\gamma -\zeta -1}\right)\left|g\left(u\left(s\right),C^{\zeta }u\left(s\right)\right)\right|ds\\ & & +|\frac{1}{\Gamma (\gamma -\zeta )}|\int_{t_{1}}^{t_{2}}{\left(t_{2}-s\right)}^{\gamma -\zeta -1}\left|g\left(u\left(s\right),C^{\zeta }u\left(s\right)\right)\right|ds+|\frac{1}{\left(1-\xi \mu^{2}\right)\Gamma \left(\gamma \right)}\\ & & \{\frac{\left(t_{2}^{2-\zeta }-t_{1}^{2-\zeta }\right)\left[a\left(1-\xi \mu^{2}\right)\Gamma \left(\eta +3\right)+2b\left(2-\xi \mu^{2}\right)\Gamma \left(\eta +4\right)\left(1-\xi \mu^{3}\right)\right]}{\Gamma \left(3-\zeta \right)[a\left(1-\xi \mu^{2}\right)\Gamma \left(\eta +3\right)+2b\left(1-\xi \mu^{3}\right)\Gamma \left(\eta +4\right)]}\\ & & +\frac{2b(1-\xi \mu^{2}\Gamma \left(\eta +4\right)\left(t_{2}^{3-\zeta }-t_{1}^{3-\zeta }\right))}{\Gamma \left(4-\zeta \right)[a\left(1-\xi \mu^{2}\right)\Gamma \left(\eta +3\right)+2b\left(2-\xi \mu^{2}\right)\left(1-\xi \mu^{3}\right)\Gamma \left(\eta +4\right)]}\}|\\ & & \int_{0}^{1}{\left(1-s\right)}^{\gamma -1}\left|g\left(u\left(s\right),C^{\zeta }u\left(s\right)\right)\right|ds\\ & & +|\{\frac{\left(1-\xi \mu^{2}\right)\Gamma \left(\eta +3\right)\Gamma \left(\eta +4\right)\left(1-\xi \mu^{3}\right)\left(t_{2}^{2-\zeta }-t_{1}^{2-\zeta }\right)}{\Gamma \left(3-\zeta \right)\Gamma \left(\gamma -1\right)[a\left(1-\xi \mu^{2}\right)\Gamma \left(\eta +3\right)+2b\left(1-\xi \mu^{3}\right)\Gamma \left(\eta +4\right)]}\\ & & +\frac{\left(1-\xi \mu^{2}\right)\Gamma \left(\eta +3\right)\Gamma \left(\eta +4\right)\left(t_{2}^{3-\zeta }-t_{1}^{3-\zeta }\right)}{\Gamma \left(4-\zeta \right)\Gamma \left(\gamma -1\right)[a\left(1-\xi \mu^{2}\right)\Gamma \left(\eta +3\right)+2b\left(1-\xi \mu^{3}\right)\Gamma \left(\eta +4\right)]}\}|\\ & & \int_{0}^{1}{\left(1-s\right)}^{\gamma -2}\left|g\left(u\left(s\right),C^{\zeta }u\left(s\right)\right)\right|+|\frac{\lambda }{\Gamma (\gamma +\eta )}\\ & & \{\frac{\left(1-\xi \mu^{2}\right)\Gamma \left(\eta +3\right)\Gamma \left(\eta +4\right)\left(1-\xi \mu^{3}\right)\left(t_{2}^{2-\zeta }-t_{1}^{2-\zeta }\right)}{\Gamma \left(3-\zeta \right)[a\left(1-\xi \mu^{2}\right)\Gamma \left(\eta +3\right)+2b\left(1-\xi \mu^{3}\right)\Gamma \left(\eta +4\right)]}\\ & & +\frac{\left(1-\xi \mu^{2}\right)\Gamma \left(\eta +3\right)\Gamma \left(\eta +4\right)\left(t_{2}^{3-\zeta }-t_{1}^{3-\zeta }\right)}{\Gamma \left(4-\zeta \right)[a\left(1-\xi \mu^{2}\right)\Gamma \left(\eta +3\right)+2b\left(1-\xi \mu^{3}\right)\Gamma \left(\eta +4\right)]}\}|\\ & & \int_{0}^{\nu }{\left(\nu -s\right)}^{\eta +\gamma -1}\left|g\left(u\left(s\right),C^{\zeta }u\left(s\right)\right)\right|ds+|\frac{\xi }{\left(1-\xi \mu^{2}\right)\Gamma \left(\gamma \right)}\\ & & \{\frac{\left[a\left(1-\xi \mu^{2}\right)\Gamma \left(\eta +3\right)+2b\left(2-\xi \mu^{2}\right)\left(1-\xi \mu^{3}\right)\Gamma \left(\eta +4\right)\right]\left(t_{2}^{2-\zeta }-t_{1}^{2-\zeta }\right)}{\Gamma \left(3-\zeta \right)[a\left(1-\xi \mu^{2}\right)\Gamma \left(\eta +3\right)+2b\left(1-\xi \mu^{3}\right)\Gamma \left(\eta +4\right)]}\\ & & +\frac{2b\left(1-\xi \mu^{2}\right)\Gamma \left(\eta +4\right)\left(t_{2}^{3-\zeta }-t_{1}^{3-\zeta }\right)}{\Gamma \left(4-\zeta \right)[a\left(1-\xi \mu^{2}\right)+2b\Gamma \left(\eta +4\right)\Gamma \left(\eta +3\right)\left(1-\xi \mu^{3}\right)]}\}|\\ & & \int_{0}^{\mu }{\left(\mu -s\right)}^{\gamma -1}\left|g\left(u\left(s\right),C^{\zeta }u\left(s\right)\right)\right|ds\\ & \leq & \frac{M}{\left|\Gamma (\gamma -\zeta +1)\right|}\left(t_{1}^{\gamma -\zeta }-t_{2}^{\gamma -\zeta }\right)+\frac{M}{\left|\Gamma (\gamma -\zeta +1)\right|}{\left(t_{2}-t_{1}\right)}^{\gamma -\zeta }\\ & & +\frac{M}{|\left(1-\xi \mu^{2}\right)\Gamma \left(\gamma +1\right)\left[a\left(1-\xi \mu^{2}\right)\Gamma \left(\eta +3\right)+2b\left(1-\xi \mu^{3}\right)\Gamma \left(\eta +4\right)\right]|}\\ & & |\{\frac{\left(t_{2}^{2-\zeta }-t_{1}^{2-\zeta }\right)\left[a\left(1-\xi \mu^{2}\right)\Gamma \left(\eta +3\right)+2b\left(2-\xi \mu^{2}\right)\left(1-\xi \mu^{3}\right)\Gamma \left(\eta +4\right)\right]}{\Gamma (3-\zeta )}\\ & & +\frac{\left(t_{2}^{3-\zeta }-t_{1}^{3-\zeta }\right)2b\left(1-\xi \mu^{2}\right)\Gamma \left(\eta +4\right)}{\Gamma (4-\zeta )}\}|+\frac{M}{\left|\Gamma (\gamma )\right|}\\ & & |\{\frac{\left(1-\xi \mu^{2}\right)\Gamma \left(\eta +3\right)\left(1-\xi \mu^{3}\right)\Gamma \left(\eta +4\right)\left(t_{2}^{2-\zeta }-t_{1}^{2-\zeta }\right)}{\Gamma \left(3-\zeta \right)[a\left(1-\xi \mu^{2}\right)\Gamma \left(\eta +3\right)+2b\left(1-\xi \mu^{3}\right)\Gamma \left(\eta +4\right)]}\\ & & +\frac{\left(1-\xi \mu^{2}\right)\Gamma \left(\eta +3\right)\Gamma \left(\eta +4\right)\left(t_{2}^{3-\zeta }-t_{1}^{3-\zeta }\right)}{\Gamma \left(4-\zeta \right)[a\left(1-\xi \mu^{2}\right)\Gamma \left(\eta +3\right)+2b\left(1-\xi \mu^{3}\right)\Gamma \left(\eta +4\right)]}\}|\\ & & +M|\frac{\lambda \nu^{\eta +\gamma }\left(1-\xi \mu^{2}\right)\Gamma \left(\eta +3\right)\Gamma \left(\eta +4\right)}{\Gamma \left(\eta +\gamma +1\right)[a\left(1-\xi \mu^{2}\right)\Gamma \left(\eta +3\right)+2b\left(1-\xi \mu^{3}\right)\Gamma \left(\eta +4\right)]}\\ & & \{\frac{\left(1-\xi \mu^{3}\right)\left(t_{2}^{2-\zeta }-t_{1}^{2-\zeta }\right)}{\Gamma (3-\zeta )}+\frac{\left(t_{2}^{3-\zeta }-t_{1}^{3-\zeta }\right)}{\Gamma (4-\zeta )}\}|+M|\frac{\xi }{\left(1-\xi \mu^{2}\right)\Gamma \left(\gamma +1\right)}\\ & & \frac{1}{\left[a\left(1-\xi \mu^{2}\right)\Gamma \left(\eta +3\right)+2b\left(1-\xi \mu^{3}\right)\Gamma \left(\eta +4\right)\right]}\\ & & \{\frac{\left(t_{2}^{2-\zeta }-t_{1}^{2-\zeta }\right)\left[a\left(1-\xi \mu^{2}\right)\Gamma \left(\eta +3\right)+2b\left(2-\xi \mu^{2}\right)\Gamma \left(\eta +4\right)\left(1-\xi \mu^{3}\right)\right]}{\Gamma (3-\zeta )}\\ & & +\frac{\left(t_{2}^{3-\zeta }-t_{1}^{3-\zeta }\right)2b\left(1-\xi \mu^{2}\right)\Gamma \left(\eta +4\right)}{\Gamma (4-\zeta )}\}|. \end{array}$$ (35)

$$\begin{array}{ccc} \|\tau u\left(t_{2}\right)-\tau u\left(t_{1}\right){\|}_{X} & \leq & M|\frac{1}{\Gamma (\gamma +1)}|\left(t_{1}^{\gamma }-t_{2}^{\gamma }\right)+M|\frac{1}{\Gamma (\gamma +1)}|{\left(t_{2}-t_{1}\right)}^{\gamma }+M|\frac{1}{\left(1-\xi \mu^{2}\right)\Gamma \left(\gamma +1\right)}\\ & & \{\frac{\left(t_{2}^{2}-t_{1}^{2}\right)\left[a\left(1-\xi \mu^{2}\right)\Gamma \left(\eta +3\right)+2b\left(2-\xi \mu^{2}\right)\Gamma \left(\eta +4\right)\left(1-\xi \mu^{3}\right)\right]}{a\left(1-\xi \mu^{2}\right)\Gamma \left(\eta +3\right)+2b\left(1-\xi \mu^{3}\right)\Gamma \left(\eta +4\right)}\\ & & +\frac{\left(t_{2}^{3}-t_{1}^{3}\right)2b\left(1-\xi \mu^{2}\right)\Gamma \left(\eta +4\right)}{a\left(1-\xi \mu^{2}\right)\Gamma \left(\eta +3\right)+2b\left(1-\xi \mu^{3}\right)}\Gamma \left(\eta +4\right)\}|\\ & & +M|\frac{\left(1-\xi \mu^{2}\right)\Gamma \left(\eta +4\right)\left(1-\xi \mu^{3}\right)\Gamma \left(\eta +3\right)\left(t_{2}^{2}-t_{1}^{2}\right)}{\Gamma \left(\gamma \right)[a\left(1-\xi \mu^{2}\right)\Gamma \left(\eta +3\right)+2b\left(1-\xi \mu^{3}\right)\Gamma \left(\eta +4\right)]}|\\ & & +M|\frac{\left(1-\xi \mu^{2}\right)\Gamma \left(\eta +3\right)\Gamma \left(\eta +4\right)\left(t_{2}^{3}-t_{1}^{3}\right)}{\Gamma \left(\gamma \right)[a\left(1-\xi \mu^{2}\right)\Gamma \left(\eta +3\right)+2b\left(1-\xi \mu^{3}\right)\Gamma \left(\eta +4\right)]}|\\ & & +M|\frac{\lambda \nu^{\eta +\gamma }\left(1-\xi \mu^{2}\right)1-\xi \mu^{3})\Gamma \left(\eta +3\right)\Gamma \left(\eta +4\right)(\left(t_{2}^{2}-t_{1}^{2}\right)}{\Gamma \left(\eta +\gamma +1\right)[a\left(1-\xi \mu^{2}\right)\Gamma \left(\eta +3\right)+2b\left(1-\xi \mu^{3}\right)\Gamma \left(\eta +4\right)]}|\\ & & +M|\lambda \frac{\nu^{\eta +\gamma }\left(1-\xi \mu^{2}\right)\Gamma \left(\eta +3\right)\Gamma \left(\eta +4\right)\left(t_{2}^{3}-t_{1}^{3}\right)}{\Gamma \left(\eta +\gamma +1\right)[a\left(1-\xi \mu^{2}\right)\Gamma \left(\eta +3\right)+2b\left(1-\xi \mu^{3}\right)\Gamma \left(\eta +4\right)]}|\\ & & +M|\frac{\xi \mu^{\gamma }\left[a\left(1-\xi \mu^{2}\right)\Gamma \left(\eta +3\right)+2b\left(2-\xi \mu^{2}\right)\left(1-\xi \mu^{3}\right)\Gamma \left(\eta +4\right)\right]\left(t_{2}^{2}-t_{1}^{2}\right)}{\left(1-\xi \mu^{2}\right)\Gamma \left(\gamma +1\right)\left[a\left(1-\xi \mu^{2}\right)\Gamma \left(\eta +3\right)+2b\left(1-\xi \mu^{3}\right)\Gamma \left(\eta +4\right)\right]}|\\ & & +M|\frac{2b\xi \mu^{\gamma }\left(1-\xi \mu^{2}\right)\Gamma \left(\eta +4\right)\left(t_{2}^{3}-t_{1}^{3}\right)}{\left(1-\xi \mu^{2}\right)\Gamma \left(\gamma +1\right)\left[a\left(1-\xi \mu^{2}\right)\Gamma \left(\eta +3\right)+2b\left(1-\xi \mu^{3}\right)\Gamma \left(\eta +4\right)\right]}|\\ & & +M|\frac{1}{\Gamma (\gamma -\zeta +1)}|\left(t_{1}^{\gamma -\zeta }-t_{2}^{\gamma -\zeta }\right)+M|\frac{1}{\Gamma (\gamma +\zeta +1)}|{\left(t_{2}-t_{1}\right)}^{\gamma -\zeta }\\ & & +M|\frac{1}{\left(1-\xi \mu^{2}\right)\Gamma \left(\gamma +1\right)\left[a\left(1-\xi \mu^{2}\right)\Gamma \left(\eta +3\right)+2b\left(1-\xi \mu^{3}\right)\Gamma \left(\eta +4\right)\right]}\\ & & \{\frac{\left(t_{2}^{2-\zeta }-t_{1}^{2-\zeta }\right)\left[a\left(1-\xi \mu^{2}\right)\Gamma \left(\eta +3\right)+2b\left(2-\xi \mu^{2}\right)\Gamma \left(\eta +4\right)\left(1-\xi \mu^{3}\right)\right]}{\Gamma (3-\zeta )}\\ & & +\frac{\left(t_{2}^{3-\zeta }-t_{1}^{3-\zeta }\right)2b\left(1-\xi \mu^{2}\right)\Gamma \left(\eta +4\right)}{\Gamma (4-\zeta )}\}|+M|\frac{1}{\Gamma (\gamma )}\\ & & \{\frac{\left(1-\xi \mu^{2}\right)\Gamma \left(\eta +3\right)\left(1-\xi \mu^{3}\right)\Gamma \left(\eta +4\right)\left(t_{2}^{2-\zeta }-t_{1}^{2-\zeta }\right)}{\Gamma \left(3-\zeta \right)[a\left(1-\xi \mu^{2}\right)\Gamma \left(\eta +3\right)+2b\left(1-\xi \mu^{3}\right)\Gamma \left(\eta +4\right)]}\\ & & +\frac{\left(1-\xi \mu^{2}\right)\Gamma \left(\eta +3\right)\Gamma \left(\eta +4\right)\left(t_{2}^{3-\zeta }-t_{1}^{3-\zeta }\right)}{\Gamma \left(4-\zeta \right)[a\left(1-\xi \mu^{2}\right)\Gamma \left(\eta +3\right)+2b\left(1-\xi \mu^{3}\right)\Gamma \left(\eta +4\right)]}\}|\\ & & +M|\frac{\lambda \nu^{\eta +\gamma }\left(1-\xi \mu^{2}\right)\Gamma \left(\eta +3\right)\Gamma \left(\eta +4\right)}{\Gamma \left(\eta +\gamma +1\right)[a\left(1-\xi \mu^{2}\right)\Gamma \left(\eta +3\right)+2b\left(1-\xi \mu^{3}\right)\Gamma \left(\eta +4\right)]}\\ & & \{\frac{\left(1-\xi \mu^{3}\right)\left(t_{2}^{2-\zeta }-t_{1}^{2-\zeta }\right)}{\Gamma (3-\zeta )}+\frac{\left(t_{2}^{3-\zeta }-t_{1}^{3-\zeta }\right)}{\Gamma (4-\zeta )}\}|+M|\frac{\xi }{\left(1-\xi \mu^{2}\right)\Gamma \left(\gamma +1\right)}\\ & & \{\frac{\left(t_{2}^{2-\zeta }-t_{1}^{2-\zeta }\right)\left[a\left(1-\xi \mu^{2}\right)\Gamma \left(\eta +3\right)+2b\left(2-\xi \mu^{2}\right)\Gamma \left(\eta +4\right)\left(1-\xi \mu^{3}\right)\right]}{\Gamma \left(3-\zeta \right)[a\left(1-\xi \mu^{2}\right)\Gamma \left(\eta +3\right)+2b\left(1-\xi \mu^{3}\right)\Gamma \left(\eta +4\right)]}\\ & & +\frac{\left(t_{2}^{3-\zeta }-t_{1}^{3-\zeta }\right)2b\left(1-\xi \mu^{2}\right)\Gamma \left(\eta +4\right)}{\Gamma \left(4-\zeta \right)[a\left(1-\xi \mu^{2}\right)\Gamma \left(\eta +3\right)+2b\left(1-\xi \mu^{3}\right)\Gamma \left(\eta +4\right)]}\}|, \end{array}$$ (36)

which shows that ‖*τu*(*t*_2_) − *τu*(*t*_1_)‖_*X*_ → 0 if *t*_2_ → *t*_1_, then by Arzela-Ascoli theorem it is concluded that *τ* is completely continuous operator.

**Step 4**: Suppose a set W={u∈IR,u=ϱτ(u),0<ϱ<1}. We have to prove that the set *W* is bounded. For this if *u* ∈ *W*, then *u* = *ϱτ*(*u*), 0 < *ϱ* < 1. For any 0 ≤ *t* ≤ 1, it can be written as:

$$\begin{array}{ccc} \frac{1}{\varrho }\left|u\left(t\right)\right| & \leq & |\frac{1}{\Gamma (\gamma )}|\int_{0}^{t}{\left(t-s\right)}^{\gamma -1}\left|g\left(u\left(s\right),C^{\zeta }u\left(s\right)\right)\right|ds\\ & & +|\frac{t^{2}}{\left[a\left(1-\xi \mu^{2}\right)\Gamma \left(\eta +3\right)+2b\left(1-\xi \mu^{3}\right)\Gamma \left(\eta +4\right)\right]}\{\frac{a\Gamma (\eta +3)}{\Gamma (\gamma )}+\frac{t}{\Gamma (\gamma )}\\ & & +\frac{2b\left(2-\xi \mu^{2}\right)\Gamma \left(\eta +4\right)\left(1-\xi \mu^{3}\right)}{\left(1-\xi \mu^{2}\right)\Gamma \left(\gamma \right)}\}|\int_{0}^{1}{\left(1-s\right)}^{\gamma -1}\left|g\left(u\left(s\right),C^{\zeta }u\left(s\right)\right)\right|ds\\ & & +|\frac{t^{2}\Gamma \left(\eta +4\right)\left(1-\xi \mu^{2}\right)\Gamma \left(\eta +3\right)\left(1-\xi \mu^{3}+t\right)}{\Gamma \left(\gamma -1\right)[a\left(1-\xi \mu^{2}\right)\Gamma \left(\eta +3\right)+2b\left(1-\xi \mu^{3}\right)\Gamma \left(\eta +4\right)]}|\\ & & \int_{0}^{1}{\left(1-s\right)}^{\gamma -2}\left|g\left(u\left(s\right),C^{\zeta }u\left(s\right)\right)ds\right|+|\frac{\lambda }{\Gamma (\gamma +\eta )}\\ & & \frac{\Gamma \left(\eta +3\right)\left(1-\xi \mu^{2}\Gamma \left(\eta +4\right)\right)\left(t^{2}\left(1-\xi \mu^{3}\right)+t^{3}\right)}{\left[a\left(1-\xi \mu^{2}\right)\Gamma \left(\eta +3\right)+2b\left(1-\xi \mu^{3}\right)\Gamma \left(\eta +4\right)\right]}|\int_{0}^{\nu }{\left(1-s\right)}^{\gamma +\eta -1}\left|g\left(u\left(s\right),C^{\zeta }u\left(s\right)\right)ds\right|\\ & & +|\frac{t^{2}\xi \left[a\left(1-\xi \mu^{2}\right)\Gamma \left(\eta +3\right)+2b\Gamma \left(\eta +4\right)\left[\left(1-\xi \mu^{3}\right)\left(2-\xi \mu^{2}\right)+t\left(1-\xi \mu^{2}\right)\right]\right]}{\left(1-\xi \mu^{2}\right)\Gamma \left(\gamma \right)\left[a\left(1-\xi \mu^{2}\right)\Gamma \left(\eta +3\right)+2b\left(1-\xi \mu^{3}\right)\Gamma \left(\eta +4\right)\right]}|\\ & & \int_{0}^{\mu }{\left(\mu -s\right)}^{\gamma -1}\left|g\left(u\left(s\right),C^{\zeta }u\left(s\right)\right)\right|ds\\ & \leq & |\frac{1}{\Gamma (\gamma +1)}|M+|\frac{t^{2}}{\left(1-\xi \mu^{2}\right)\Gamma \left(\gamma +1\right)}\\ & & \{\frac{a\left(1-\xi \mu^{2}\right)\Gamma \left(\eta +3\right)+2b\Gamma \left(\eta +4\right)\left[\left(1-\xi \mu^{3}\right)\left(2-\xi \mu^{2}\right)+t\left(1-\xi \mu^{2}\right)\right]}{\left[a\left(1-\xi \mu^{2}\right)\Gamma \left(\eta +3\right)+2b\left(1-\xi \mu^{3}\right)\Gamma \left(\eta +4\right)\right]}\}|M\\ & & +|\frac{t^{2}\Gamma \left(\eta +4\right)\Gamma \left(\eta +3\right)\left(1-\xi \mu^{3}+t\right)\left(1-\xi \mu^{2}\right)}{\Gamma \left(\gamma \right)[a\left(1-\xi \mu^{2}\right)\Gamma \left(\eta +3\right)+2b\left(1-\xi \mu^{3}\right)\Gamma \left(\eta +4\right)]}|M\\ & & +|\frac{\lambda \nu^{\gamma +\eta }\Gamma \left(\eta +3\right)\left(1-\xi \mu^{2}\right)\Gamma \left(\eta +4\right)\left(t^{2}\left(1-\xi \mu^{3}\right)+t^{3}\right)}{\Gamma \left(\gamma +\eta +1\right)[a\left(1-\xi \mu^{2}\right)\Gamma \left(\eta +3\right)+2b\left(1-\xi \mu^{3}\right)\Gamma \left(\eta +4\right)]}|M\\ & & +|\frac{\xi \mu^{\gamma }t^{2}\left\{a\left(1-\xi \mu^{2}\right)\Gamma \left(\eta +3\right)+2b\Gamma \left(\eta +4\right)\left[\left(1-\xi \mu^{3}\right)\left(2-\xi \mu^{2}\right)+t\left(1-\xi \mu^{2}\right)\right]\right\}}{\left(1-\xi \mu^{2}\right)\Gamma \left(\gamma +1\right)\left[a\left(1-\xi \mu^{2}\right)\Gamma \left(\eta +3\right)+2b\left(1-\xi \mu^{3}\right)\Gamma \left(\eta +4\right)\right]}|M\\ & = & M[|\frac{1}{\Gamma (\gamma +1)}|+|\frac{t^{2}}{\left(1-\xi \mu^{2}\right)\Gamma \left(\gamma +1\right)}\\ & & \left\{\frac{a\left(1-\xi \mu^{2}\right)\Gamma \left(\eta +3\right)+2b\Gamma \left(\eta +4\right)\left[\left(1-\xi \mu^{3}\right)\left(2-\xi \mu^{2}\right)+t\left(1-\xi \mu^{2}\right)\right]}{\left[a\left(1-\xi \mu^{2}\right)\Gamma \left(\eta +3\right)+2b\left(1-\xi \mu^{3}\right)\Gamma \left(\eta +4\right)\right]}\}\right|\\ & & +|\frac{t^{2}\Gamma \left(\eta +4\right)\Gamma \left(\eta +3\right)\left(1-\xi \mu^{3}+t\right)\left(1-\xi \mu^{2}\right)}{\Gamma \left(\gamma \right)\left[a\left(1-\xi \mu^{2}\right)\Gamma \left(\eta +3\right)+2b\left(1-\xi \mu^{3}\right)\right]\Gamma \left(\eta +4\right)}|\\ & & +|\frac{\lambda \nu^{\gamma +\eta }\Gamma \left(\eta +4\right)\;\left(1-\xi \mu^{2}\right)Gamma\left(\eta +3\right)\left(t^{2}\left(1-\xi \mu^{3}\right)+t^{3}\right)}{\Gamma \left(\gamma +\eta +1\right)[a\left(1-\xi \mu^{2}\right)\Gamma \left(\eta +3\right)+2b\left(1-\xi \mu^{3}\right)\Gamma \left(\eta +4\right)]}|\\ & & +|\frac{\xi \mu^{\gamma }t^{2}\left\{a\left(1-\xi \mu^{2}\right)\Gamma \left(\eta +3\right)+2b\Gamma \left(\eta +4\right)\left[\left(1-\xi \mu^{3}\right)\left(2-\xi \mu^{2}\right)+t\left(1-\xi \mu^{2}\right)\right]\right\}}{\left(1-\xi \mu^{2}\right)\Gamma \left(\gamma +1\right)\left[a\left(1-\xi \mu^{2}\right)\Gamma \left(\eta +3\right)+2b\right)\left(1-\xi \mu^{3}\right)\Gamma \left(\eta +4\right]}|], \end{array}$$ (37)

which implies,

$$\begin{array}{ccc} \frac{1}{\varrho }\left|u\left(t\right)\right| & \leq & MP_{1}. \end{array}$$

Therefore,

$$\begin{array}{c} \|y\|\leq \varrho (MP_{1}). \end{array}$$ (38)

and,

$$\begin{array}{ccc} \frac{1}{\varrho }\left|C^{\zeta }\tau u\left(t\right)\right| & \leq & |\frac{1}{\Gamma (\gamma -\zeta )}|\int_{0}^{t}{\left(t-s\right)}^{\gamma -\zeta -1}\left|g\left(u\left(s\right),C^{\zeta }u\left(s\right)\right)\right|ds\\ & & +|\frac{1}{\left(1-\xi \mu^{2}\right)\Gamma \left(\gamma \right)\{a\left(1-\xi \mu^{2}\right)\Gamma \left(\eta +3\right)+2b\left(1-\xi \mu^{3}\right)\Gamma \left(\eta +4\right)\}}\\ & & \{\frac{t^{2-\zeta }\left[a\left(1-\xi \mu^{2}\right)\Gamma \left(\eta +3\right)+2b\left(2-\xi \mu^{2}\right)\Gamma \left(\eta +4\right)\left(1-\xi \mu^{3}\right)\right]}{\Gamma (3-\zeta )}\\ & & +\frac{t^{3-\zeta }2b\left(1-\xi \mu^{2}\right)\Gamma \left(\eta +4\right)}{\Gamma (4-\zeta )}\}|\int_{0}^{1}{\left(1-s\right)}^{\gamma -1}\left|g\left(u\left(s\right),C^{\zeta }u\left(s\right)\right)\right|ds\\ & & +|\frac{\left(1-\xi \mu^{2}\right)\Gamma \left(\eta +3\right)\Gamma \left(\eta +4\right)}{\Gamma \left(\gamma -1\right)[a\left(1-\xi \mu^{2}\right)\Gamma \left(\eta +3\right)+2b\left(1-\xi \mu^{3}\right)\Gamma \left(\eta +4\right)]}\\ & & \{\frac{\left(1-\xi \mu^{3}\right)t^{2-\zeta }}{\Gamma (3-\zeta )}+\frac{t^{3-\zeta }}{\Gamma (4-\zeta |)}\}|\int_{0}^{1}{\left(1-s\right)}^{\gamma -2}\left|g\left(u\left(s\right),C^{\zeta }u\left(s\right)\right)\right|ds\\ & & +|\frac{\lambda \left(1-\xi \mu^{2}\right)\Gamma \left(\eta +3\right)\Gamma \left(\eta +4\right)}{\Gamma \left(\eta +\gamma \right)[a\left(1-\xi \mu^{2}\right)\Gamma \left(\eta +3\right)+2b\Gamma \left(\eta +4\right)\left(1-\xi \mu^{3}\right)]}\\ & & \{\frac{t^{2-\zeta }\left(1-\xi \mu^{3}\right)}{\Gamma (3-\zeta )}+\frac{t^{3-\zeta }}{\Gamma (4-\zeta )}\}|\int_{0}^{\nu }{\left(\nu -s\right)}^{\gamma +\eta -1}\left|g\left(u\left(s\right),C^{\zeta }u\left(s\right)\right)\right|ds\\ & & +|\frac{\xi }{\Gamma \left(\gamma \right)\left(1-\xi \mu^{2}\right)\left[a\left(1-\xi \mu^{2}\right)\Gamma \left(\eta +3\right)+2b\left(1-\xi \mu^{3}\right)\Gamma \left(\eta +4\right)\right]}\\ & & \{\frac{t^{2-\zeta }\left[a\left(1-\xi \mu^{2}\right)\Gamma \left(\eta +3\right)+2b\left(2-\xi \mu^{2}\right)\Gamma \left(\eta +4\right)\left(1-\xi \mu^{3}\right)\right]}{\Gamma (3-\zeta )}\\ & & +\frac{2b\left(1-\xi \mu^{2}\right)\Gamma \left(\eta +4\right)t^{3-\zeta }}{\Gamma (4-\zeta )}\}|\int_{0}^{\mu }{\left(\mu -s\right)}^{\gamma -1}\left|g\left(u\left(s\right),C^{\zeta }u\left(s\right)\right)\right|ds \end{array}$$ (39)

$$\begin{array}{ccc} & \leq & |\frac{1}{\Gamma (\gamma -\zeta +1)}|M+|\frac{1}{\left(1-\xi \mu^{2}\right)\Gamma \left(\gamma +1\right)}\\ & & \frac{1}{\left\{a\left(1-\xi \mu^{2}\right)\Gamma \left(\eta +3\right)+2b\left(1-\xi \mu^{3}\right)\Gamma \left(\eta +4\right)\right\}}\{\frac{t^{2-\zeta }a\left(1-\xi \mu^{2}\right)\Gamma \left(\eta +3\right)}{\Gamma (3-\zeta )}\\ & & +\frac{2bt^{2-\zeta }\left(2-\xi \mu^{2}\right)\Gamma \left(\eta +4\right)\left(1-\xi \mu^{3}\right)}{\Gamma (3-\zeta )}+\frac{2t^{3-\zeta }b\left(1-\xi \mu^{2}\right)\Gamma \left(\eta +4\right)}{\Gamma (4-\zeta )}\}|M\\ & & +|\frac{\left(1-\xi \mu^{2}\right)\Gamma \left(\eta +3\right)\Gamma \left(\eta +4\right)}{\Gamma \left(\gamma \right)[a\Gamma \left(\left(1-\xi \mu^{2}\right)\eta +3\right)+2b\left(1-\xi \mu^{3}\right)\Gamma \left(\eta +4\right)]}\{\frac{t^{2-\zeta }\left(1-\xi \mu^{3}\right)}{\Gamma (3-\zeta )}+\frac{t^{3-\zeta }}{\Gamma (4-\zeta |)}\}|M\\ & & +|\frac{\lambda \nu^{\eta +\gamma }\left(1-\xi \mu^{2}\right)\Gamma \left(\eta +3\right)\Gamma \left(\eta +4\right)}{\Gamma \left(\eta +\gamma +1\right)[a\left(1-\xi \mu^{2}\Gamma \left(\eta +3\right)\right)+2b\left(1-\xi \mu^{3}\right)\Gamma \left(\eta +4\right)]}\{\frac{t^{2-\zeta }\left(1-\xi \mu^{3}\right)}{\Gamma (3-\zeta )}\\ & & +\frac{t^{3-\zeta }}{\Gamma (4-\zeta )}\}|M+|\frac{\xi \mu^{\gamma }}{\left(1-\xi \mu^{2}\right)\Gamma \left(\gamma +1\right)\left[a\left(1-\xi \mu^{2}\right)\Gamma \left(\eta +3\right)+2b\left(1-\xi \mu^{3}\right)\Gamma \left(\eta +4\right)\right]}\\ & & \{\frac{t^{2-\zeta }\left[a\left(1-\xi \mu^{2}\right)\Gamma \left(\eta +3\right)+2b\left(2-\xi \mu^{2}\right)\left(1-\xi \mu^{3}\right)\Gamma \left(\eta +4\right)\right]}{\Gamma (3-\zeta )}\\ & & +\frac{2b\left(1-\xi \mu^{2}\right)\Gamma \left(\eta +4\right)t^{3-\zeta }}{\Gamma (4-\zeta )}\}|M\\ & = & M\{|\frac{1}{\Gamma (\gamma -\zeta +1)}|+|\frac{1}{\left(1-\xi \mu^{2}\right)\Gamma \left(\gamma +1\right)}\\ & & \frac{1}{\left\{\left(1-\xi \mu^{2}\right)a\Gamma \left(\eta +3\right)+2b\left(1-\xi \mu^{3}\right)\Gamma \left(\eta +4\right)\right\}}\{\frac{t^{2-\zeta }\left[a\left(1-\xi \mu^{2}\right)\Gamma \left(\eta +3\right)\right]}{\Gamma (3-\zeta )}\\ & & +\frac{2bt^{2-\zeta }\left(2-\xi \mu^{2}\right)\Gamma \left(\eta +4\right)\left(1-\xi \mu^{3}\right)}{\Gamma (3-\zeta )}+\frac{2t^{3-\zeta }b\left(1-\xi \mu^{2}\right)\Gamma \left(\eta +4\right)}{\Gamma (4-\zeta )}\}|\\ & & +|\frac{\left(1-\xi \mu^{2}\right)\Gamma \left(\eta +3\right)\Gamma \left(\eta +4\right)}{\Gamma \left(\gamma \right)[a\left(1-\xi \mu^{2}\right)\Gamma \left(\eta +3\right)+2b\left(1-\xi \mu^{3}\right)\Gamma \left(\eta +4\right)]}\{\frac{t^{2-\zeta }\left(1-\xi \mu^{3}\right)}{\Gamma (3-\zeta )}+\frac{t^{3-\zeta }}{\Gamma (4-\zeta |)}\}|\\ & & +|\frac{\lambda \nu^{\eta +\gamma }\left(1-\xi \mu^{2}\right)\Gamma \left(\eta +3\right)\Gamma \left(\eta +4\right)}{\Gamma \left(\eta +\gamma +1\right)[a\left(1-\xi \mu^{2}\right)\Gamma \left(\eta +3\right)+2b\left(1-\xi \mu^{3}\right)\Gamma \left(\eta +4\right)]}\{\frac{t^{2-\zeta }\left(1-\xi \mu^{3}\right)}{\Gamma (3-\zeta )}\\ & & +\frac{t^{3-\zeta }}{\Gamma (4-\zeta )}\}|+|\frac{\xi \mu^{\gamma }}{\left(1-\xi \mu^{2}\right)\Gamma \left(\gamma +1\right)\left[a\left(1-\xi \mu^{2}\right)\Gamma \left(\eta +3\right)+2b\left(1-\xi \mu^{3}\right)\Gamma \left(\eta +4\right)\right]}\\ & & \{\frac{t^{2-\zeta }\left[a\left(1-\xi \mu^{2}\right)\Gamma \left(\eta +3\right)+2b\left(2-\xi \mu^{2}\right)\left(1-\xi \mu^{3}\right)\Gamma \left(\eta +4\right)\right]}{\Gamma (3-\zeta )}\\ & & +\frac{2b\Gamma \left(\eta +4\right)\left(1-\xi \mu^{2}\right)t^{3-\zeta }}{\Gamma (4-\zeta )}\}|\}. \end{array}$$ (40)

This implies,

$$\begin{array}{ccc} \frac{1}{\varrho }\left|C^{\zeta }\tau u\left(t\right)\right| & \leq & MP_{2}, \end{array}$$ (41)

therefore,

$$\begin{array}{c} |C^{\zeta }\tau u\left(t\right)|\leq \varrho \left(MP_{2}\right). \end{array}$$ (42)

Thus, from Eqs (38) and (42)

$$\begin{array}{ccc} {\left\|u\right\|}_{X} & \leq & \varrho \left(MP_{1}\right)+\varrho \left(MP_{2}\right)\\ {\left\|u\right\|}_{X} & \leq & \varrho M(P_{1}+P_{2}). \end{array}$$ (43)

Since 0 < *ϱ* < 1, so,

$$\begin{array}{c} {\left\|\tau \left(u\right)\right\|}_{X}<\infty . \end{array}$$ (44)

This shows that *W* is bounded.

By schaefer fixed point theorem, BVP (1) has at least one solution.

**Example 1**: Consider the following fractional problem:

$$\begin{array}{c} \left\{ \begin{array}{c} C^{\frac{7}{2}}u\left(t\right)=\frac{u\left(t\right)e^{-2\pi }}{20(2e^{t}+\sqrt{\pi })}+\frac{C^{\frac{1}{2}}u\left(t\right)}{16\sqrt{\pi }\left(2e^{t}+\pi \right)\left(C^{\frac{1}{2}}u\left(t\right)\right)+4},\;\;\;t\in \left[0,1\right]\\ u\left(0\right)=0,\;\;u^{\prime }\left(0\right)=0,\;\;u\left(1\right)=\frac{4}{5}y\left(\frac{1}{2}\right),\;\;u^{\prime }\left(1\right)=\frac{2}{3}J^{\frac{3}{4}}y\left(\frac{3}{5}\right)\;\;\; \end{array}\right. \end{array}$$ (45)

$$\begin{array}{c} g\left(u,v\right)=\frac{ue^{-2\pi }}{20(2e^{t}+\sqrt{\pi })}+\frac{v}{16\sqrt{\pi }\left(2e^{t}+\pi \right)\left(z+4\right)}. \end{array}$$ (46)

Let $u,u_{1},v,v_{1}\in I\;R$ and *t* ∈ [0, 1]. Then

$$\begin{array}{c} |g\left(u,v\right)-g\left(u_{1},v_{1}\right)=\left|\frac{\left(u-u_{1}\right)e^{-2\pi }}{20(2e^{t}+\sqrt{\pi })}+\frac{v-v_{1}}{16\sqrt{\pi }\left(2e^{t}+\pi \right)\left(v-v_{1}+4\right)}\right|. \end{array}$$ (47)

$$\begin{array}{c} |g\left(u,v\right)-g\left(u_{1},v_{1}\right)\leq |\frac{e^{-2\pi }}{20(2e^{t}+\sqrt{\pi })}\left|\right|\left(u-u_{1}\right)\left|+\right|\frac{1}{16\sqrt{\pi }\left(2e^{t}+\pi \right)\left(v-v_{1}\right)+4}||v-v_{1}| \end{array}$$ (48)

$$\begin{array}{ccc} & \leq & |\frac{e^{-2\pi }}{20(2e^{t}+\sqrt{\pi })}\left|\right|\left(u-u_{1}\right)\left|+\right|\frac{1}{16\sqrt{\pi }\left(2e^{t}+\pi \right)}||v-v_{1}|. \end{array}$$ (49)

Here,

$$\begin{array}{c} \theta =\frac{e^{-2\pi }}{20(2e^{t}+\sqrt{\pi })},\;\;\;\;\;\;\vartheta =\frac{1}{16\sqrt{\pi }\left(2e^{t}+\pi \right)}. \end{array}$$ (50)

$$\begin{array}{c} \theta =0.00002079,\;\;\;\;\;\;\vartheta =0.0041106. \end{array}$$ (51)

$$\begin{array}{ccc} P_{1} & = & |\frac{1}{\Gamma (\gamma +1)}|+|\frac{t^{2}}{\left(1-\mu^{2}\xi \right)\Gamma \left(\gamma +1\right)}\\ & & \left\{\frac{\left[a\left(1-\mu^{2}\xi \right)\Gamma \left(\eta +3\right)+2b\left\{\left(2-\xi \mu^{2}\right)\left(1-\xi \mu^{3}\right)+t\left(1-\xi \mu^{2}\right)\right\}\Gamma \left(\eta +4\right)\right]}{\left[a\Gamma \left(\eta +3\right)\left(1-\xi \mu^{2}\right)+2b\Gamma \left(\eta +4\right)\left(1-\xi \mu^{3}\right)\right]}\}\right|\\ & & +|\frac{t^{2}\Gamma \left(\eta +3\right)\Gamma \left(\eta +4\right)\left(1-\xi \mu^{2}\right)(1+t-\mu^{3}\xi }{\Gamma \left(\gamma \right)[a\left(1-\xi \mu^{2}\right)\Gamma \left(\eta +3\right)+2b\left(1-\xi \mu^{3}\right)\Gamma \left(\eta +4\right)]}|\\ & & +|\frac{\nu^{\gamma +\eta }\lambda \Gamma \left(\eta +3\right)\Gamma \left(\eta +4\right)\left(1-\xi \mu^{2}\right)\left(t^{2}\left(1-\xi \mu^{3}\right)+t^{3}\right)}{\Gamma \left(\gamma +\eta +1\right)[a\Gamma \left(\eta +3\right)\left(1-\xi \mu^{2}\right)+2b\Gamma \left(\eta +4\right)\left(1-\xi \mu^{3}\right)]}|\\ & & +|\frac{\mu^{\gamma }\xi t^{2}\left[a\left(1-\xi \mu^{2}\right)\Gamma \left(\eta +3\right)+2b\left\{\left(2-\xi \mu^{2}\right)\left(1-\xi \mu^{3}\right)+t\left(1-\xi \mu^{2}\right)\right\}\Gamma \left(\eta +4\right)\right]}{\Gamma \left(\gamma +1\right)\left(1-\xi \mu^{2}\right)\left[a\Gamma \left(\eta +3\right)\left(1-\xi \mu^{2}\right)+2b\Gamma \left(\eta +4\right)\left(1-\xi \mu^{3}\right)\right]}|. \end{array}$$ (52)

$$\begin{array}{ccc} P_{2} & = & |\frac{1}{\Gamma (\gamma -\zeta +1)}|+|\frac{1}{\Gamma \left(\gamma +1\right)\left(1-\xi \mu^{2}\right)}\\ & & \frac{1}{a\Gamma \left(\eta +3\right)\left(1-\xi \mu^{2}\right)+2b\Gamma \left(\eta +4\right)\left(1-\xi \mu^{3}\right)}\{\frac{t^{2-\zeta }\left[a\Gamma \left(\eta +3\right)\left(1-\xi \mu^{2}\right)\right]}{\Gamma (3-\zeta )}\\ & & +\frac{2b\left(1-\xi \mu^{3}\right)\left(2-\xi \mu^{2}\right)\Gamma \left(\eta +4\right)t^{2-\zeta }}{\Gamma (3-\zeta )}+\frac{2bt^{3-\zeta }\left(1-\xi \mu^{2}\right)\Gamma \left(\eta +4\right)}{\Gamma (4-\zeta )}\}|\\ & & +|\frac{\left(1-\xi \mu^{2}\right)\Gamma \left(\eta +3\right)\Gamma \left(\eta +4\right)}{\Gamma \left(\gamma \right)[a\left(1-\xi \mu^{2}\right)\Gamma \left(\eta +3\right)+2b\left(1-\xi \mu^{3}\right)\Gamma \left(\eta +4\right)]}\{\frac{\left(1-\xi \mu^{3}\right)t^{2-\zeta }}{\Gamma (3-\zeta )}+\frac{t^{3-\zeta }}{\Gamma (4-\zeta )}\}|\\ & & +|\frac{\nu^{\gamma +\eta }\lambda \left(1-\xi \mu^{2}\right)\Gamma \left(\eta +3\right)\Gamma \left(\eta +4\right)}{\Gamma \left(\eta +\gamma +1\right)[a\Gamma \left(\eta +3\right)\left(1-\xi \mu^{2}\right)+2b\left(1-\xi \mu^{3}\right)\Gamma \left(\eta +4\right)]}\{\frac{t^{2-\zeta }\left(1-\xi \mu^{3}\right)}{\Gamma (3-\zeta )}\\ & & +\frac{t^{3-\zeta }}{\Gamma (4-\zeta )}\}|+|\frac{\xi \mu^{\gamma }}{\left(1-\xi \mu^{2}\right)\Gamma \left(\gamma +1\right)\left[a\left(1-\xi \mu^{2}\right)\Gamma \left(\eta +3\right)+2b\left(1-\xi \mu^{3}\right)\Gamma \left(\eta +4\right)\right]}\\ & & \{\frac{t^{2-\zeta }\left[a\left(1-\xi \mu^{2}\right)\Gamma \left(\eta +3\right)+2b\left(2-\xi \mu^{2}\right)\left(1-\xi \mu^{3}\right)\Gamma \left(\eta +4\right)\right]}{\Gamma (3-\zeta )}\\ & & +\frac{2b\left(1-\xi \mu^{2}\right)\Gamma \left(\eta +4\right)t^{3-\zeta }}{\Gamma (4-\zeta )}\}|. \end{array}$$ (53)

By putting *γ* = 7/2, *ζ* = 1/2, *η* = 3/4, *ξ* = 4/5, *ν* = 3/5, *μ*1/2, λ = 2/3, in *P*_1_ and *P*_2_, we have *P*_1_ = 1.927765 and *P*_2_ = 1.131351.

This implies to

$$\begin{array}{c} \left(P_{1}+P_{2}\right)\left(\theta +\vartheta \right)=\left(1.927765+1.131351\right)\left(0.00002079+0.0041106\right)=0.01<1. \end{array}$$ (54)

Hence, by Theorem (4.1), the BVP (1) has a unique solution.

**Example 2**: Consider the following boundary value problem:

$$\begin{array}{c} \left\{ \begin{array}{c} C^{\frac{7}{2}}u\left(t\right)=2t^{2}cosu\left(t\right)+tsinC^{\frac{1}{2}}u\left(t\right),\;\;\;t\in \left[0,1\right],\\ u\left(0\right)=0,\;\;u^{\prime }\left(0\right)=0,\;\;u\left(1\right)=\frac{4}{5}y\left(\frac{1}{2}\right),\;\;u^{\prime }\left(1\right)=\frac{2}{3}J^{\frac{3}{4}}y\left(\frac{3}{5}\right).\;\;\; \end{array}\right. \end{array}$$ (55)

**Step 1**: It is obvious that the operator *τ* is continuous because the function |*g*(*u*, *v*)| = |2*t*^2^*cosu* + *tsinv*| is continuous function.

**Step 2**: In this step we will prove that the operator *τ* maps bounded sets into bounded sets in X:

$$\begin{array}{c} |g\left(u,v\right)|=|2t^{2}cosu+tsinv|\leq 3=M. \end{array}$$ (56)

By putting the values of *γ* = 7/2, *ζ* = 1/2, *η* = 3/4, *ξ* = 4/5, *ν* = 3/5, *μ*1/2, λ = 2/3, in *P*_1_ and *P*_2_, we have *P*_1_ = 1.927765 and *P*_2_ = 1.131351.

Now put the values of *M* and *P*_1_ in Eq 27, we have

$$\begin{array}{ccc} \left|\tau u(t)\right| & \leq & MP_{1}\\ & = & \left(3)(1.927765\right)\\ & = & 5.783295\\ \left|\tau u(t)\right| & < & \infty . \end{array}$$ (57)

Now put the values of *M* and *P*_2_ in Eq 30, we have

$$\begin{array}{ccc} |C^{\frac{1}{2}}\tau u\left(t\right)| & \leq & MP_{2}\\ & = & \left(3)(1.131351\right)\\ & = & 3.394053\\ |C^{\frac{1}{2}}\tau u\left(t\right)| & < & \infty . \end{array}$$ (58)

Therefore,

$$\begin{array}{c} {\left\|\tau \left(u\right)\right\|}_{X}\leq M\left(P_{1}+P_{2}\right)=10.977348. \end{array}$$

This implies to

$$\begin{array}{c} {\left\|\tau \left(u\right)\right\|}_{X}<\infty . \end{array}$$ (59)

Hence, *τ* is bounded.

**Step 3**: In this step we will prove that the operator *τ* is equi-continuous on [0, 1]:

‖*τu*(*t*_2_) − *τu*(*t*_1_)‖_*X*_ ≤

$$\begin{array}{ccc} & & M|\frac{1}{\Gamma (\gamma +1)}|\left(t_{1}^{\gamma }-t_{2}^{\gamma }\right)+M|\frac{1}{\Gamma (\gamma +1)}|{\left(t_{2}-t_{1}\right)}^{\gamma }+M|\frac{1}{\left(1-\xi \mu^{2}\right)\Gamma \left(\gamma +1\right)}\\ & & \{\frac{\left(t_{2}^{2}-t_{1}^{2}\right)\left[a\left(1-\xi \mu^{2}\right)\Gamma \left(\eta +3\right)+2b\left(2-\xi \mu^{2}\right)\Gamma \left(\eta +4\right)\left(1-\xi \mu^{3}\right)\right]}{a\left(1-\xi \mu^{2}\right)\Gamma \left(\eta +3\right)+2b\left(1-\xi \mu^{3}\right)\Gamma \left(\eta +4\right)}\\ & & +\frac{\left(t_{2}^{3}-t_{1}^{3}\right)2b\left(1-\xi \mu^{2}\right)\Gamma \left(\eta +4\right)}{a\left(1-\xi \mu^{2}\right)\Gamma \left(\eta +3\right)+2b\left(1-\xi \mu^{3}\right)}\Gamma \left(\eta +4\right)\}|\\ & & +M|\frac{\left(1-\xi \mu^{2}\right)\Gamma \left(\eta +4\right)\left(1-\xi \mu^{3}\right)\Gamma \left(\eta +3\right)\left(t_{2}^{2}-t_{1}^{2}\right)}{\Gamma \left(\gamma \right)[a\left(1-\xi \mu^{2}\right)\Gamma \left(\eta +3\right)+2b\left(1-\xi \mu^{3}\right)\Gamma \left(\eta +4\right)]}|\\ & & +M|\frac{\left(1-\xi \mu^{2}\right)\Gamma \left(\eta +3\right)\Gamma \left(\eta +4\right)\left(t_{2}^{3}-t_{1}^{3}\right)}{\Gamma \left(\gamma \right)[a\left(1-\xi \mu^{2}\right)\Gamma \left(\eta +3\right)+2b\left(1-\xi \mu^{3}\right)\Gamma \left(\eta +4\right)]}|\\ & & +M|\frac{\lambda \nu^{\eta +\gamma }\left(1-\xi \mu^{2}\right)1-\xi \mu^{3})\Gamma \left(\eta +3\right)\Gamma \left(\eta +4\right)(\left(t_{2}^{2}-t_{1}^{2}\right)}{\Gamma \left(\eta +\gamma +1\right)[a\left(1-\xi \mu^{2}\right)\Gamma \left(\eta +3\right)+2b\left(1-\xi \mu^{3}\right)\Gamma \left(\eta +4\right)]}|\\ & & +M|\lambda \frac{\nu^{\eta +\gamma }\left(1-\xi \mu^{2}\right)\Gamma \left(\eta +3\right)\Gamma \left(\eta +4\right)\left(t_{2}^{3}-t_{1}^{3}\right)}{\Gamma \left(\eta +\gamma +1\right)[a\left(1-\xi \mu^{2}\right)\Gamma \left(\eta +3\right)+2b\left(1-\xi \mu^{3}\right)\Gamma \left(\eta +4\right)]}|\\ & & +M|\frac{\xi \mu^{\gamma }\left[a\left(1-\xi \mu^{2}\right)\Gamma \left(\eta +3\right)+2b\left(2-\xi \mu^{2}\right)\left(1-\xi \mu^{3}\right)\Gamma \left(\eta +4\right)\right]\left(t_{2}^{2}-t_{1}^{2}\right)}{\left(1-\xi \mu^{2}\right)\Gamma \left(\gamma +1\right)\left[a\left(1-\xi \mu^{2}\right)\Gamma \left(\eta +3\right)+2b\left(1-\xi \mu^{3}\right)\Gamma \left(\eta +4\right)\right]}|\\ & & +M|\frac{2b\xi \mu^{\gamma }\left(1-\xi \mu^{2}\right)\Gamma \left(\eta +4\right)\left(t_{2}^{3}-t_{1}^{3}\right)}{\left(1-\xi \mu^{2}\right)\Gamma \left(\gamma +1\right)\left[a\left(1-\xi \mu^{2}\right)\Gamma \left(\eta +3\right)+2b\left(1-\xi \mu^{3}\right)\Gamma \left(\eta +4\right)\right]}|\\ & & +M|\frac{1}{\Gamma (\gamma -\zeta +1)}|\left(t_{1}^{\gamma -\zeta }-t_{2}^{\gamma -\zeta }\right)+M|\frac{1}{\Gamma (\gamma +\zeta +1)}|{\left(t_{2}-t_{1}\right)}^{\gamma -\zeta }\\ & & +M|\frac{1}{\left(1-\xi \mu^{2}\right)\Gamma \left(\gamma +1\right)\left[a\left(1-\xi \mu^{2}\right)\Gamma \left(\eta +3\right)+2b\left(1-\xi \mu^{3}\right)\Gamma \left(\eta +4\right)\right]}\\ & & \{\frac{\left(t_{2}^{2-\zeta }-t_{1}^{2-\zeta }\right)\left[a\left(1-\xi \mu^{2}\right)\Gamma \left(\eta +3\right)+2b\left(2-\xi \mu^{2}\right)\Gamma \left(\eta +4\right)\left(1-\xi \mu^{3}\right)\right]}{\Gamma (3-\zeta )}\\ & & +\frac{\left(t_{2}^{3-\zeta }-t_{1}^{3-\zeta }\right)2b\left(1-\xi \mu^{2}\right)\Gamma \left(\eta +4\right)}{\Gamma (4-\zeta )}\}|+M|\frac{1}{\Gamma (\gamma )}\\ & & \{\frac{\left(1-\xi \mu^{2}\right)\Gamma \left(\eta +3\right)\left(1-\xi \mu^{3}\right)\Gamma \left(\eta +4\right)\left(t_{2}^{2-\zeta }-t_{1}^{2-\zeta }\right)}{\Gamma \left(3-\zeta \right)[a\left(1-\xi \mu^{2}\right)\Gamma \left(\eta +3\right)+2b\left(1-\xi \mu^{3}\right)\Gamma \left(\eta +4\right)]}\\ & & +\frac{\left(1-\xi \mu^{2}\right)\Gamma \left(\eta +3\right)\Gamma \left(\eta +4\right)\left(t_{2}^{3-\zeta }-t_{1}^{3-\zeta }\right)}{\Gamma \left(4-\zeta \right)[a\left(1-\xi \mu^{2}\right)\Gamma \left(\eta +3\right)+2b\left(1-\xi \mu^{3}\right)\Gamma \left(\eta +4\right)]}\}|\\ & & +M|\frac{\lambda \nu^{\eta +\gamma }\left(1-\xi \mu^{2}\right)\Gamma \left(\eta +3\right)\Gamma \left(\eta +4\right)}{\Gamma \left(\eta +\gamma +1\right)[a\left(1-\xi \mu^{2}\right)\Gamma \left(\eta +3\right)+2b\left(1-\xi \mu^{3}\right)\Gamma \left(\eta +4\right)]}\\ & & \{\frac{\left(1-\xi \mu^{3}\right)\left(t_{2}^{2-\zeta }-t_{1}^{2-\zeta }\right)}{\Gamma (3-\zeta )}+\frac{\left(t_{2}^{3-\zeta }-t_{1}^{3-\zeta }\right)}{\Gamma (4-\zeta )}\}|+M|\frac{\xi }{\left(1-\xi \mu^{2}\right)\Gamma \left(\gamma +1\right)}\\ & & \{\frac{\left(t_{2}^{2-\zeta }-t_{1}^{2-\zeta }\right)\left[a\left(1-\xi \mu^{2}\right)\Gamma \left(\eta +3\right)+2b\left(2-\xi \mu^{2}\right)\Gamma \left(\eta +4\right)\left(1-\xi \mu^{3}\right)\right]}{\Gamma \left(3-\zeta \right)[a\left(1-\xi \mu^{2}\right)\Gamma \left(\eta +3\right)+2b\left(1-\xi \mu^{3}\right)\Gamma \left(\eta +4\right)]}\\ & & +\frac{\left(t_{2}^{3-\zeta }-t_{1}^{3-\zeta }\right)2b\left(1-\xi \mu^{2}\right)\Gamma \left(\eta +4\right)}{\Gamma \left(4-\zeta \right)[a\left(1-\xi \mu^{2}\right)\Gamma \left(\eta +3\right)+2b\left(1-\xi \mu^{3}\right)\Gamma \left(\eta +4\right)]}\}|. \end{array}$$ (60)

It is clear from the above equation that every term has *t*_2_ − *t*_1_ so if *t*_2_ → *t*_1_ then ‖*τu*(*t*_2_) − *τu*(*t*_1_)‖_*X*_ → 0. By Arzela-Ascoli theorem it is concluded that *τ* equi-continuous operator.

**Step 4**: Suppose a set W={u∈IR,u=ϱτ(u),0<ϱ<1}. We have to prove that the set W is bounded. For this if *u* ∈ *W*, then *u* = *ϱτ*(*u*), 0 < *ϱ* < 1. For any 0 ≤ *t* ≤ 1, it can be written as,

$$\begin{array}{ccc} {\left\|u\right\|}_{X} & \leq & \varrho M(P_{1}+P_{2}). \end{array}$$ (61)

Let *ϱ* = 0.5 then

$$\begin{array}{ccc} {\left\|u\right\|}_{X} & \leq & (0.5)(3)(1.927765+1.131351)=4.588674. \end{array}$$ (62)

This shows that *W* is bounded. By Theorem (4.2), BVP (1) has at least one solution.

## 5 Comparative analysis

The authors in [42] investigated boundary value problems for FEDs with multiple orders of fractional integrals and derivatives. The orders of derivatives and integrals belongs to the interval [1, 2] in both FEDs and boundary conditions. Existence and uniqueness results are obtained using Sadovskii’s fixed point theorem and Banach’s contraction mapping principle.

Cona and Bal in [43] established a model consists of fractional order Integral-Differential equation with some suitable initial conditions. The order of fractional order Integral-Differential equation was belongs to [0, 1] in that model. They used fixed point method to find the sufficient conditions for existence and uniqueness of considered problem.

The authors in [44] presented the coupled system of integro-differential equations with periodic and nonlocal integral boundary conditions. They proved the existence and uniqueness of their presented model with the help of fixed point theorems. They use two different order Caputo Fractional derivative in their coupled system, the order of one derivative is belongs to [2, 3] and the other is belongs to [1, 2].

Sharif, Hamood and Ghadle gave some new investigations into the existence and uniqueness analysis of the fractional Volterra-Fredholm integro differential equations of order [0, 1] with beginning conditions that are nonlinear Caputo fractional equations. The desired outcomes are demonstrated using the fractional inequality, a variation of the nonlinear alternative of Leray-Schauder in Banach spaces and the Banach fixed point theorem for non-self mappings. Additionally, by using the contraction mapping principle, the uniqueness results were established. They investigated the existence and uniqueness of solutions for the absence of research on nonlinear fractional Volterra-Fredholm integro-differential equations with initial conditions [45].

In parallel with all these most recent studies, we investigate existence and uniqueness for the solution of Caputo-fractional differential equation of order [3, 4] involving implicit function with well-posed boundary conditions. Our boundary conditions are well-posed which mean the solution behaviour changes continuously with boundary condition and solution is not highly sensitive to changes in final data. Solution of well-posed boundary condition problem can be deducted on a computer using stable algorithm.

## 6 Mathematical contribution

In this article we concluded mathematically that if a function is continuous and a positive real number *M* exists such that |*g*(*t*, *u*, *v*)| ≤ *M* for all *u*, *v* ∈ *X* and for each *t* ∈ [0, 1] then solution of the considered problem exist. Moreover if (*P*_1_ + *P*_2_)(*θ* + *ϑ*) < 1 then the considered BVP has a unique solution and for the applicability of these two points we take examples where *γ* = 7/2, *ζ* = 1/2, *η* = 3/4, *ξ* = 4/5, *ν* = 3/5, *μ*1/2, λ = 2/3, and value of (*P*_1_+ *P*_2_)(*θ* + *ϑ*) become less than 1 and |*g*(*t*, *u*, *v*)| became less to *M*. So our considered model exist and have a unique solution.

## 7 Conclusion

We managed to employ Banach contraction principle and Schaefer fixed point theorem to study the existence and uniqueness of solution for a well-possed implicit fractional boundary value problem involving Caputo derivatives of order *γ* ∈ [3, 4] and *ζ* ∈ [1, 2]. Results of this paper give a different opinion about the differentiation of derivative in the discussed boundary value problem. Considered function is necessarily third-order differentiable to guarantee the existence of solution could be forth order differentiable. If the function is not third order differentiable then the solution *u*(*t*) is not exist for q = 4 because *d*^4^/*dx*^4^ does not exist.

The model under study is generalized version of many recent studies. We use some examples to demonstrate the results. In future this model will help to convert forth order differential problems in fractional differential models. The main advantage of this conversion is fractional differential models are more accurate as compare to ordinary differential model because it preserves the history of procedure and predict the future.

## References

[1] Akraami MH and Erjaee GH. Existence, uniqueness and well-posed conditions on a class of fractional differential equations with boundary conditions, Journal of fractional calculus and application, 2015; 6(2): 171–185.

[2] Granas A and Dugundji J. Fixed point theory, Springer Monographs in Mathematics, Springer-Verlag, New York, (2003).

[3] Podlubny I. Fractional Differential Equations. New York, Academic Press, 1999.

[4] Tu ST, Nisshimoto and Jaw S. Applications of fractional calculus to ordinary and partial differential equations of second order, Hiroshima Mathematical Journal, 1993; 23: 63–77. doi: 10.32917/hmj/1206128378

[5] Metzler F, Schick W, Kilian HG and Nonnenmacher TF. Relaxation in filled polymers: A fractional calculus approach, Journal of Chemical Physics 1995; 103: 7180–7186. doi: 10.1063/1.470346

[6] Glockle WG and Nonnenmacher TF. A fractional calculus approach of self-similar protein dynamics, Biophysical Journal, 1995; 68: 46–53. doi: 10.1016/S0006-3495(95)80157-8 7711266 PMC1281659

[7] Rahimy M. Applications of Fractional Differential Equations, Applied Mathematical Sciences, 2010; 4(50): 2453–2461.

[8] Chen W, Liu W, Liang H, Jiang M, and Dai Z. Response of storm surge and M2 tide to typhoon speeds along coastal Zhejiang Province. Ocean Engineering, 2023; 270, 113646. doi: 10.1016/j.oceaneng.2023.113646

[9] Xie X, Xie B, Cheng J, Chu Q, and Dooling T. A simple Monte Carlo method for estimating the chance of a cyclone impact. Natural Hazards, 2021; 107(3), 2573–2582. doi: 10.1007/s11069-021-04505-2

[10] Rousan AA, Ayoub NY, Alzoubi FY, Khateeb H, Al-Qadi M, Hasan MK, et al. A fractional LC-RC circuit, An international journal of for theory and applications, 2006; 9(1).

[11] Kilbas AA, Srivastava HM and Trujillo JJ. Theory and Applications of Fractional Differential Equations, North-Holland Mathematics Studies, Elsevier Science Amsterdam, 2006; 204.

[12] Ahmad B, Alsaedi A and Assolami A. Existence results for Caputo type fractional differential equations with four-point nonlocal fractional integral boundary conditions, Electronic Journal of Qualitative Theory of Differential Equations, 2012; 93: 1–11. doi: 10.14232/ejqtde.2012.1.93

[13] Benchohra M and Ouaar F. Existence results for nonlinear fractional differential equations with integral boundary conditions, Mathematical Analysis and Applications, 2010; 2(4): 7–15.

[14] Ntouyas SK. Existence results for first order boundary value problems for fractional differential equations and inclusions with fractional integral boundary conditions, Journal of Fractional Calculus and Applications, 2012; 3(9): 1–14.

[15] Chai G. Existence results of positive solutions for boundary value problems of fractional differential equations, Boundary Value Problems, 2013; 109.

[16] Ahmad B and Nieto JJ, Riemann-Liouville fractional integro-differential equations with fractional nonlocal integral boundary conditions, Boundary Value Problems, 2011; 36.

[17] Zhonga W and Lin W. Nonlocal and multiple-point boundary value problem for fractional differential equations, Computers and Mathematics with Applications, 2010; 3(59): 1345–1351. doi: 10.1016/j.camwa.2009.06.032

[18] Lakoud AG and Khaldi R. Solvability of a fractional boundary value problem with fractional integral condition, Nonlinear Analysis: Theory, Methods and Applications, 2012; 75(4):2692–2700. doi: 10.1016/j.na.2011.11.014

[19] Choudhary S, and Daftardar-Gejji V. Nonlinear multi-order fractional differential equation with periodic/ anti-periodic boundary conditions, International Journal for Theory and Applications, 2014; 17(2): 333–345.

[20] Akram G and Jurrat B. Solvability of Three Point Fractional Boundary Value Problem Using Mittag-Leffler Function, International Journal of Applied and Computational Mathematics, 2023; 9(3): 44. doi: 10.1007/s40819-023-01516-4

[21] Dutta PN, and Choudhury BS. A generalisation of contraction principle in metric spaces. Fixed Point Theory and Applications, 2008;1: 406368. doi: 10.1155/2008/406368

[22] Badawi H, Shawagfeh N, and Arqub OA. Fractional conformable stochastic integrodifferential equations: existence, uniqueness, and numerical simulations utilizing the shifted Legendre spectral collocation algorithm. Mathematical Problems in Engineering, 2022. doi: 10.1155/2022/5104350

[23] Sweis H, Arqub OA, and Shawagfeh N. Fractional delay integrodifferential equations of nonsingular kernels: existence, uniqueness, and numerical solutions using Galerkin algorithm based on shifted Legendre polynomials. International Journal of Modern Physics C, 2023; 34(04): 2350052. doi: 10.1142/S0129183123500523

[24] Sweis H, Shawagfeh N, and Arqub OA. Fractional crossover delay differential equations of Mittag-Leffler kernel: Existence, uniqueness, and numerical solutions using the Galerkin algorithm based on shifted Legendre polynomials. Results in Physics, 2022; 41, 105891. doi: 10.1016/j.rinp.2022.105891

[25] Arqub OA., Mezghiche R, and Maayah B. Fuzzy M-fractional integrodifferential models: theoretical existence and uniqueness results, and approximate solutions utilizing the Hilbert reproducing kernel algorithm. Frontiers in Physics, 2023; 11, 1252919. doi: 10.3389/fphy.2023.1252919

[26] Shi M, Hu W, Li M, Zhang J, Song X, and Sun W. Ensemble regression based on polynomial regression-based decision tree and its application in the in-situ data of tunnel boring machine. Mechanical Systems and Signal Processing, 2023; 188, 110022. doi: 10.1016/j.ymssp.2022.110022

[27] li TAA, Xiao Z, Jiang H, and Li B. A Class of Digital Integrators Based on Trigonometric Quadrature Rules. IEEE Transactions on Industrial Electronics, 2024; 71(6), 6128–6138. doi: 10.1109/TIE.2023.3290247

[28] Goufo EFD, Ravichandran C and Birajdar GA. Self-similarity techniques for chaotic attractors with many scrolls using step series switching, Mathematical Modelling and Analysis, 2021; 26(4): 591–611. doi: 10.3846/mma.2021.13678

[29] Ravichandran C, Jothimani K, Nisar KS, Mahmoud EE, and Yahia IS. An interpretation on controllability of Hilfer fractional derivative with nondense domain, Alexandria Engineering Journal, 2022; 61(12): 9941–9948. doi: 10.1016/j.aej.2022.03.011

[30] Ravichandran C, Munusamy K, Nisar KS and Valliammal N. Results on neutral partial integro-differential equations using Monch-Krasnosel’Skii fixed point theorem with nonlocal conditions, Fractal and Fractional, 2022; 6(2): 75. doi: 10.3390/fractalfract6020075

[31] Jothimani K, Valliammal N, Alsaeed S, Nisar K S, and Ravichandran C. Controllability Results of Hilfer Fractional Derivative Through Integral Contractors, Qualitative Theory of Dynamical Systems, 2023; 22(4): 137. doi: 10.1007/s12346-023-00833-9

[32] Nisar KS, Logeswari K, Ravichandran C and Sabarinathan S. New frame of fractional neutral ABC-derivative with IBC and mixed delay, Chaos, Solitons and Fractals, 2023; 175: 114050. doi: 10.1016/j.chaos.2023.114050

[33] Nisar KS, Jagatheeshwari R, Ravichandran C and Veeresha P. An effective analytical method for fractional Brusselator reaction–diffusion system. Mathematical Methods in the Applied Sciences, 2023; 46(18): 18749–18758. doi: 10.1002/mma.9589

[34] Nisar KS, Alsaeed S, Kaliraj K, Ravichandran C, Albalawi W and Abdel-Aty AH. Existence criteria for fractional differential equations using the topological degree method, AIMS Mathematics, 2023; 8(9): 21914–21928. doi: 10.3934/math.20231117

[35] Bedi P, Kumar A, Abdeljawad T and Khan A. Study of Hilfer fractional evolution equations by the properties of controllability and stability, Alexandria Engineering Journal, 2021; 60(4): 3741–3749. doi: 10.1016/j.aej.2021.02.014

[36] Bedi P, Kumar A, Abdeljawad T, Khan ZA and Khan A. Existence and approximate controllability of Hilfer fractional evolution equations with almost sectorial operators. Advances in Difference Equations, 2020; 1: 1–15.

[37] Jiang B, Zhao Y, Dong J, and Hu J. Analysis of the influence of trust in opposing opinions: An inclusiveness-degree based Signed Deffuant–Weisbush model. Information Fusion, 2024; 104, 102173. doi: 10.1016/j.inffus.2023.102173

[38] Shi Y, Song C, Chen Y, Rao H, and Yang T. Complex standard eigenvalue problem derivative computation for laminar–turbulent transition prediction. AIAA Journal, 2023; 61(8), 3404–3418. doi: 10.2514/1.J062212

[39] Houas M and Benbachir M. Existence solutions for three point boundary value problem for differential equations, Journal of Fractional Calculus and Applications, 2015;6(1): 160–174.

[40] Wang Y. Some fourth order differential equations modeling suspension bridges, 2016.

[41] Zhang S. Positive solutions for boundary-value problems of nonlinear fractional diffrential equations, Electronic Journal of Differential Equations 2006; 36: 1–12.

[42] Kherraz T, Benbachir M, Lakrib M, Samei ME, Kaabar MK, and Bhanotar SA. Existence and uniqueness results for fractional boundary value problems with multiple orders of fractional derivatives and integrals, chaos, Solitons and Fractals, 2023; 166: 113007. doi: 10.1016/j.chaos.2022.113007

[43] Lale CONA and Esmahan BAL. On Existence and Uniqueness of Some Fractional Order Integro-Differential Equation, Erzincan University Journal of Science and Technology, 2023;16(2): 297–310.

[44] Alruwaily Y, Aljoudi S, Almaghamsi L, Ben Makhlouf A, and Alghamdi N. Existence and uniqueness results for different orders coupled system of fractional integro-differential equations with anti-periodic nonlocal integral boundary conditions, Symmetry, 2023; 15(1): 182. doi: 10.3390/sym15010182

[45] Sharif AA., Hamood MM, and Ghadle KP. An investigation on the existence and uniqueness analysis of the fractional voltera-fredholm integrio-differential equations, Bulletin of the International Mathemstical Virtual Institute, 2023; 13(2): 313–326.

